# From Pan-T-Cell Depletion to Selective Graft Engineering: Progress, Limitations, and Open Questions

**DOI:** 10.3390/jcm15218407

**Published:** 2026-10-28

**Authors:** Kirsty Sharplin, Sorur Moshgelgosha, Gurman Kaur

**Affiliations:** 1 Therapeutic Apheresis Service, Cell, Apheresis and Gene Therapies, NHS Blood and Transplant, Oxford OX3 9BQ, UK; kirsty.sharplin@nhsbt.nhs.uk; 2 Oxford University Hospitals Transplant Unit, Churchill Hospital, Oxford OX3 7LE, UK; 3 Cellular and Molecular Therapies, Cell, Apheresis and Gene Therapies, NHS Blood and Transplant, Oxford OX3 9BQ, UK; sorur.moshgelgosha@nhsbt.nhs.uk; 4 Nuffield Division of Clinical Laboratory Sciences, Radcliffe Department of Medicine, University of Oxford, Oxford OX3 9DU, UK

**Keywords:** allogeneic haematopoietic stem cell transplantation, precision graft engineering, graft-versus-host disease, graft-versus-leukaemia, post-transplant cyclophosphamide, T-cell depletion

## Abstract

The success of allogeneic haematopoietic stem cell transplantation is limited by three linked problems: graft-versus-host disease (GVHD), relapse, and infection. Pan-T-cell depletion, introduced in the 1980s, controlled GVHD but at the cost of relapse and impaired immune reconstitution. This trade-off has driven the field toward selective graft engineering, manipulating the donor graft’s cellular composition to separate GVHD-causing cells from those responsible for graft-versus-leukaemia (GVL) activity and infection control. This review traces that evolution, from CD34^+^ selection through αβ/CD19 depletion, naïve (CD45RA^+^) T-cell depletion, adoptive add-back, and gene-edited donor grafts, evaluated against current in vivo prophylaxis, principally post-transplant cyclophosphamide (PTCy). Selective graft-engineering platforms have achieved low rates of chronic GVHD and non-relapse mortality in defined settings. More recently, TREGZI (developed as Orca-T) received U.S. Food and Drug Administration (FDA) approval in June 2026 for adults with haematological malignancies undergoing matched-donor myeloablative allo-HSCT, following a randomised phase III trial against tacrolimus/methotrexate rather than PTCy. However, evidence for most other platforms remains dominated by single-arm, paediatric, or single-centre series; adult data are sparse, and no randomised trial has directly compared ex vivo graft engineering with optimised PTCy-based prophylaxis. Manufacturing infrastructure, cost, and access further limit adoption relative to established prophylaxis. Randomised trials are needed to determine whether, and for which patients, selective graft engineering offers a survival advantage.

## 1. Introduction

Haematopoietic stem cell transplantation (HSCT) remains a curative option for both high-risk haematological conditions and non-malignant disorders. However, long-term success is constrained by three principal failure modes: graft-versus-host disease (GVHD), disease relapse, and infection [1,2,3,4]. GVHD is a feared complication of transplantation, owing to the high fatality rate of early and severe forms of the disease [5]. In the longer term, chronic GVHD (cGVHD) contributes to late mortality and to significant impairment of quality of life [6].

Since the first transplants in 1968 [7,8], understanding of the immune mechanisms governing both graft-versus-leukaemia (GVL) and GVHD has evolved considerably, though it remains incomplete. Separating potentially fatal GVHD from potentially curative GVL remains a central challenge in transplant medicine. Early attempts to prevent GVHD through T-cell depletion included ex vivo incubation of donor bone marrow with anti-T-cell globulin, as reported by Rodt, Thierfelder and colleagues in the late 1970s and early 1980s [9,10]. These studies established an early clinical precedent for manipulation of the donor graft to reduce alloreactivity. Subsequent pan-T-cell depletion (TCD) strategies effectively prevented GVHD but markedly increased relapse and graft rejection, with prolonged immune deficiency and a high rate of infectious death [11,12,13]. Contemporary practice has favoured in vivo pharmacological modulation of donor-recipient alloreactivity, using serotherapy with anti-thymocyte globulin (ATG) or alemtuzumab (Campath), or chemotherapeutic post-transplant cyclophosphamide (PTCy), usually with additional immunosuppression [14]. Unlike ex vivo graft engineering, these approaches are administered to the recipient rather than directly modifying the cellular composition of the donor graft.

The risk of GVHD rises with the degree of genetic disparity between donor and recipient, which is why histocompatibility matching is fundamental to donor selection. The human leukocyte antigen (HLA) system is central to donor selection in allogeneic HSCT. An HLA-identical sibling has historically been regarded as the preferred donor where available, while a well-matched unrelated donor provides an established alternative. High-resolution typing technology allows better-matched donors to be identified; however, this does not address inequity of access. The likelihood of identifying a fully matched donor ranges from 75% among white patients of European descent to 16% among black patients of South or Central American descent [15], leaving patients from ethnic minorities with less common HLA types disproportionately reliant on mismatched donors [16].

Where fully matched donors cannot be found, mismatched donors remain an option; however, outcomes for mismatched unrelated donor (MMUD) transplants historically carried high rates of acute and cGVHD. Post-transplant cyclophosphamide (PTCy) substantially reduced these complications and has made such transplants widely practicable [17,18], although the survival disadvantage of HLA mismatch persists even in PTCy-treated transplants [19]. In a series of 17,292 unrelated transplants performed in 2016–2020, class I mismatching remained associated with inferior overall survival (OS) (HR, 1.36 [99% CI, 1.24 to 1.49]; *p* < 0.001) [19]. PTCy improved GVHD-free relapse-free survival (GRFS) when compared to conventional GVHD prophylaxis in the matched setting (HR, 0.77 [0.66 to 0.90]; *p* < 0.001). However, in MMUD transplantation, mortality remained higher than with fully matched transplants even in patients receiving PTCy (HR 1.32 [1.04 to 1.68]; *p* = 0.003), similar to patients not receiving PTCy (interaction *p* = 0.43) [19]. Reducing GVHD has therefore only taken MMUD transplantation so far, and closing the remaining survival gap may be where more selective graft engineering has something to offer.

Selective graft engineering asks a different question: can the graft itself be manipulated so that the cells responsible for GVHD are removed while those mediating GVL and immune reconstitution are retained? This review traces the evolution from CD34^+^ selection and pan-T-cell depletion through selective depletion of TCRαβ^+^ and naïve CD45RA^+^ T cells and defined cellular add-back, before considering gene-edited donor grafts and related post-transplant cellular strategies. These approaches are evaluated in the context of contemporary in vivo prophylaxis.

For the purposes of this review, selective graft engineering refers primarily to the deliberate ex vivo manipulation of the donor haematopoietic graft before infusion to alter defined cell populations while preserving those considered beneficial for engraftment, immune recovery or GVL activity. This is distinct from in vivo pharmacological GVHD prophylaxis, which acts through treatment of the recipient without directly modifying the graft. We also discuss post-transplant adoptive cellular therapies and genetically modified cell products where these extend the same biological principle, but distinguish these from manipulation of the primary transplant graft. Gene-edited donor haematopoietic cells are therefore considered an emerging form of graft engineering, whereas engineered immune-cell products administered separately after transplantation are treated as related investigational cellular therapies rather than established graft-engineering platforms. Throughout the review, ‘ex vivo graft manipulation’ is used as a broader synonym for processing of the donor graft before infusion; ‘selective depletion’ refers to removal of defined cellular subsets while retaining other graft components; and ‘cellular add-back’ refers to deliberate reintroduction of defined donor cell populations, either as part of graft reconstruction or as a separately administered post-transplant cellular intervention.

## 2. Defining Graft Engineering

**Figure 1:**
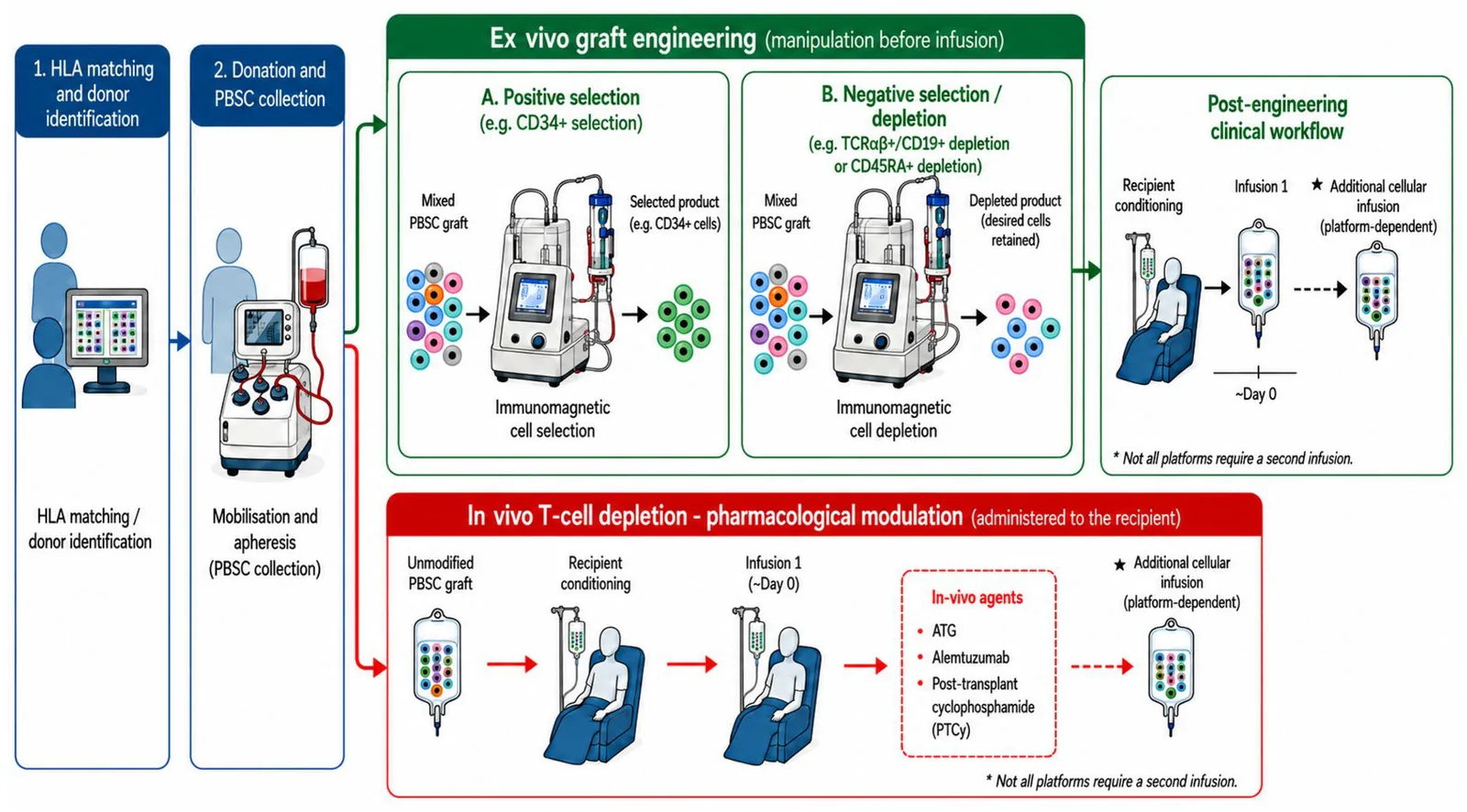
**Position of ex vivo graft engineering within the allogeneic HSCT pathway.** Following donor identification and PBSC collection, grafts may undergo ex vivo manipulation before infusion using positive-selection or negative-depletion strategies. Positive selection enriches a desired cell population, whereas negative selection removes defined cell populations while retaining other graft components. By contrast, in vivo pharmacological T-cell modulation is administered to the recipient rather than directly manipulating the graft. Solid arrows indicate steps common to all platforms and dashed arrows indicate optional platform-dependent steps.

For the vast majority, the primary source of stem cells is from peripheral blood of donors mobilised with granulocyte colony-stimulating factor (G-CSF). Following collection, these cells are either infused directly or undergo what is termed ‘graft engineering’. Graft engineering refers to the deliberate manipulation of the stem cell product before infusion to alter its cellular composition. Also referred to as ex vivo manipulation, it differs from in vivo pharmacological GVHD prophylaxis, which modulates donor-recipient alloreactivity through treatment of the recipient without directly altering the cellular composition of the graft (Figure 1).

Two strategies in graft engineering exist (Figure 1). Positive selection retains the population of interest and discards everything else, so a CD34-selected graft will deliver CD34+ cells with little else. Negative selection removes the population driving the unwanted effect, e.g., GVHD, and retains the remainder, so an αβ-depleted graft will still contain other T cell subsets, such as TCRγδ^+^ T cells. Modern technologies rely on immunomagnetic separation in closed, automated systems, principally the Miltenyi CliniMACS Plus and CliniMACS Prodigy, which reduce manual handling and improve reproducibility for GMP-compliant manufacture, and have made increasingly selective strategies such as αβ/CD19 depletion feasible in routine practice [20].

More recently, efforts to produce a more precise cellular composition have led to the term ‘precision-engineered graft’, which involves a combination of both positive and negative selection. A graft-engineered product, TREGZI (developed clinically as Orca-T), received U.S. FDA approval on 30 June 2026 for use in matched-donor HSCT with a myeloablative preparative regimen, for haematopoietic and immunologic reconstitution and to improve chronic GVHD (cGVHD)-free survival in adults with haematological malignancies [21]. The product comprises defined donor HSPC, regulatory T-cell (Treg) and conventional T-cell (Tcon) components administered sequentially as part of the transplant procedure [21,22]. Comparable work in Europe, infusing donor Treg and Tcon cells separately, has also demonstrated promising results [23,24], which we cover in detail in later sections of this review.

Target cell populations for ex vivo manufacture focus on two categories: haematopoietic stem cells, necessary for engraftment, and lymphocyte populations responsible for GVL/GVHD and immune reconstitution. Naïve T cells, identified by CD45RA, are the principal mediators of GVHD [25], whereas memory T cells retain recall responses against viruses and tumour antigens while causing substantially less GVHD in preclinical models [26]. γδ T cells recognise their targets without classical MHC restriction and are not thought to be major mediators of GVHD. Natural killer (NK) cells may contribute to GVL through KIR–ligand mismatch, particularly in acute myeloid leukaemia (AML) [27]. Tregs suppress alloreactivity and form the basis of Treg/Tcon protocols [23,24]. CD19^+^ B cells are removed in CD19 depletion protocols to reduce the risk of Epstein-Barr virus-driven post-transplant lymphoproliferative disease [28].

A separate branch of graft engineering, which addresses cell dose rather than immune composition, is expansion technology. Cord blood has been the focus of this approach, as although it tolerates HLA mismatch, it is limited by low numbers of progenitor cells and therefore risks delayed engraftment. Ex vivo expansion has been developed using both Notch ligand-mediated expansion [29] and small molecules in culture, including nicotinamide [30], StemRegenin-1 [31], and UM171 [32]. Such expanded products result in comparable engraftment rates to those of adult stem cells [31,33,34]. Although these platforms modify the quantity of the graft, they do not by themselves address the separation of GVHD from GVL.

Compared with in vivo TCD, ex vivo graft engineering is costly. Alongside the direct costs of manufacture, organisational infrastructure is required to uphold good manufacturing practice (GMP) standards, and the selection technologies themselves carry substantial capital and consumable costs. Programmes delivering engineered cell products further require the quality systems and personnel needed to support ongoing regulatory requirements. The consequences of this for expertise, service configuration and equity of access are considered in the later sections.

The principal selective graft-engineering and related strategies are summarised in Table 1 according to their cellular target, transplant setting, graft source, accompanying GVHD prophylaxis and maturity of clinical evidence. Randomised evidence is available for CD34^+^ selection [35] and TREGZI (Orca-T) [22], whereas most other selective graft-engineering platforms are supported predominantly by prospective single-arm, retrospective, registry or early-phase studies. Detailed trial-level characteristics and outcomes are provided in Supplementary Table S1. The sections that follow examine each platform in greater detail, beginning with the earliest approach, positive selection of the CD34^+^ graft. 

## 3. Positive Selection and the CD34^+^ Graft 

Positive selection of CD34^+^ cells enriches haematopoietic stem and progenitor cells while reducing the accompanying T-cell content. It was developed to address two major challenges in HLA-haploidentical stem cell transplantation (haplo-HSCT): graft rejection and severe GVHD. Early TCD approaches effectively reduced GVHD, but their application in patients with acute leukaemia was limited by high rates of graft rejection [62,63]. Subsequently, increasing the dose of haematopoietic progenitor cells, known as the “megadose” concept, was explored as a strategy to improve engraftment [64,65,66]. The Perugia group used selection of CD34^+^ cells to provide megadoses of progenitor cells while achieving extensive T-cell depletion [13]. In this study of 43 patients with high-risk acute leukaemia, engraftment was achieved in all recipients, with no cases of cGVHD and only two cases of aGVHD despite the absence of post-transplant GVHD prophylaxis [13].

Despite the favourable engraftment and GVHD outcomes, CD34^+^ positive selection resulted in extensive depletion of mature donor immune cells, which compromised post-transplant immune function [36,67,68]. Although extensive TCD effectively minimised GVHD, it also removed mature lymphocyte populations responsible for early antimicrobial defence, resulting in delayed immune reconstitution. This biological constraint translated into substantial infectious morbidity and mortality. In the extended Perugia experience of 104 patients with high-risk acute leukaemia, 38 patients died from non-leukaemic causes, of whom 27 died from infection-related complications; cytomegalovirus (CMV) alone accounted for 14 deaths [36]. This association was also evident in subsequent comparative experience, with infection accounting for 41% of deaths following CD34-selected transplantation compared with 18% after unmodified transplantation [67]. Despite these limitations, encouraging outcomes were observed in patients transplanted in remission, with 2-year event-free survival (EFS) of 48% ± 8% in AML and 46% ± 10% in ALL [36]. However, the high infectious morbidity associated with profound T-cell depletion highlighted the need for graft-engineering strategies that could control alloreactivity whilst preserving protective immune function [68,69].

Although CD34^+^ positive selection played an important role in the development of graft engineering, its current role in transplantation is limited. In the randomised BMT CTN 1301 trial, CD34-selected grafts reduced moderate-to-severe cGVHD without increasing relapse, but this advantage did not translate into an improvement in the primary cGVHD/relapse-free survival (CRFS) endpoint [35]. One-year CRFS was 60.2% with CD34^+^ selection, compared with 60.3% with PTCy and 52.6% with tacrolimus/methotrexate [35]. More importantly, the CD34^+^ selection arm showed lower OS mainly due to higher transplant-related mortality (TRM) from infections and organ failure [35,70]. Centre for International Blood and Marrow Transplant Research (CIBMTR) data show that the current use of CD34^+^ selection is limited, accounting for only 1–3% of HLA-matched related and unrelated donor transplants and ~2% of all peripheral blood stem-cell (PBSC) grafts, with most procedures performed in a small number of experienced centres [70].

Outside of graft engineering, CD34^+^ selection retains specialised clinical applications. CD34-selected stem-cell boosts are used for poor graft function after allogeneic transplantation, with a systematic review of seven studies involving 209 patients reporting complete and overall response rates of ~72% and 80%, respectively. However, most of the available evidence was retrospective, and prospective comparative studies are still lacking [71]. Therefore, although CD34^+^ selection is no longer widely used as a routine GVHD-prevention strategy, it continues to have a role in selected clinical settings.

## 4. Selective αβ^+^ T-Cell and CD19^+^ B-Cell Depletion

Another approach to GVHD reduction has been depletion of TCRαβ^+^ T cells alongside CD19^+^ B cell depletion to prevent development of post-transplant lymphoproliferative disorder (PTLD). Using the closed-system CliniMACS platform, Schumm et al. achieved GMP-compliant depletion of αβ^+^ T cells (4.7 log) and B cells (4.1 log), while retaining CD34^+^ HSCs, γδ^+^ T cells, NK cells and CD14^+^ cells within the graft [38].

This dual-depletion approach has largely been applied to haplo-HSCT, thereby expanding and expediting the treatment options available for patients. A clinical proof of concept of this approach was demonstrated in the non-malignant paediatric setting by Bertaina et al., who performed haplo-HSCT in 23 children with heterogeneous non-malignant disorders, where no patients received any post-transplantation GVHD prophylaxis. With a median follow-up of 18 months, 21/23 children were alive and disease-free (2-year DFS 91.1%); 3/23 developed skin-only grade I-II aGVHD, and no patient developed visceral aGVHD or cGVHD [43]. In a multicentre phase I/II study of 60 recipients (30 adults and 30 children) across 12 centres, acute and chronic GVHD were classified using the 1994 consensus and 2014 NIH criteria, respectively. Six of 60 patients developed grade II aGVHD (10.1% cumulative incidence), with no grade III-IV cases; among 48 patients who had engrafted and were alive at day 100, the cumulative incidence of moderate/severe cGVHD was 20.9%. There were no cases of PTLD reported. CMV reactivation occurred in 46% of recipients, while adenovirus DNAemia was more frequent in children (58%) [20].

The largest dataset for TCRαβ/CD19 cell–depleted haplo-HSCT has been reported in the paediatric setting, with the initial trial including 80 children [72] and the final analysis providing longer-term data from 213 children with acute leukaemia [39]. This study reported outcomes broadly comparable with matched unrelated donor transplantation, although this was a non-randomised comparison confined to children. Projected 10-year DFS was 71.6%, and 5-year non-relapse mortality (NRM) was 5.2%. The reported cumulative incidences of grade II-IV aGVHD and cGVHD were 14.7% and 8.1%, respectively [39]. Viral infection, including CMV, adenovirus and herpesvirus, was reported in a high proportion of patients (61.9%), and disease relapse was the main cause of treatment failure. In multivariable analysis, disease status at transplantation, pre-HSCT measurable residual disease and use of total body irradiation were independently associated with DFS [39]. TCRαβ/CD19 depletion has also been used in unrelated donor transplantation [40,41].

Earlier studies demonstrated the feasibility of CD3/CD19 depletion in haplo-HSCT in adults [46,47], although larger studies remain limited. More recently, a single-centre retrospective analysis of 122 adults receiving myeloablative conditioning, ATG, and a TCRαβ/CD19-depleted matched related or unrelated allograft found lower rates of extensive cGVHD and improved GRFS compared with T-cell-replete cohorts. Severe aGVHD was numerically lower, but the comparison was non-randomised, and the effect of graft depletion cannot be separated from other differences in the transplant regimen [42].

The immunological rationale for TCRαβ/CD19 depletion is compelling but not yet matched by the degree of clinical proof. Removing αβ T cells depletes a major alloreactive T-cell compartment while retaining γδ T cells and NK cells. In the multicentre study, these populations recovered first, with γδ T cells predominating over the first four weeks and their repertoire diversifying during early recovery, whereas conventional CD4+ and CD8+ T-cell reconstitution was slower and approached normal levels only by approximately 12 months [20]. Early γδ T and NK cell recovery therefore does not equate to complete adaptive immune reconstitution.

The infection data make the same point. CMV reactivation occurred in 46% of recipients, confined to seropositive donor-recipient pairs (D−R+ or D+R+) with no events in seronegative recipients, and two adults developed CMV disease. Adenovirus DNAemia occurred in 36% overall, more frequently in children, with adenovirus-associated disease reported in both age groups [20]. Reactivation and clinically significant disease are therefore distinct endpoints, and both depend on serostatus, ATG exposure and concomitant immunosuppression as much as on graft composition. Similarly, the evidence for an antileukaemic advantage remains uncertain; no randomised comparison has shown reduced relapse or improved survival, relapse remained the principal cause of treatment failure in the largest paediatric leukaemia series [39], and the adult study reporting improved GRFS was retrospective and single-centre [42]. Although TCRαβ/CD19 cell–depletion is a reproducible graft-engineering platform with encouraging GVHD outcomes, factors such as viral reactivation, incomplete early adaptive immune recovery and relapse remain important limitations. Naïve (CD45RA^+^) T cell depletion offers an alternative subset-selective strategy; instead of depleting the entire αβ^+^ T cell compartment, it targets specifically the naïve T cells thought to drive the alloreactivity.

## 5. Naïve (CD45RA^+^) T-Cell Depletion: Subset-Selective GVHD Prophylaxis

CD45RA^+^ depletion was developed as a more selective strategy based on the functional differences between naïve and memory T-cell subsets [48]. Naïve T cells contain a higher frequency of clones capable of recognising recipient minor histocompatibility antigens (miHA) and have a greater potential to induce severe GVHD than memory T cells [73,74]. In contrast, memory T cells retain responses against previously encountered pathogens and generally have lower GVHD potential [48,75]. Selective removal of naïve T cells therefore provides a more targeted approach to T-cell depletion, aiming to reduce severe alloreactivity while preserving the early immune protection provided by donor memory T cells.

To facilitate this approach, CD45RA was selected as the depletion target because it is expressed by naïve T cells but absent from most memory T-cell populations [48]. However, a subset of CD34^+^ haematopoietic progenitor cells also expresses CD45RA, meaning that direct CD45RA depletion from an unmanipulated apheresis product could result in the loss of these progenitor cells [48]. To overcome this, a two-step process was developed involving positive selection of CD34^+^ cells, followed by immunomagnetic depletion of CD45RA^+^ cells from the CD34-negative fraction [25,48]. This approach was evaluated in a first-in-human study of 35 patients with high-risk acute leukaemia, in which all recipients engrafted, and 2-year OS was 78%. cGVHD developed in 3/35 patients; the 2-year cumulative-incidence estimate was 9%, with death without GVHD treated as a competing risk [25]. However, 23/35 patients (66%) developed biopsy-confirmed grade II-IV aGVHD, including 3/35 (9%) with grade III and no grade IV disease; most cases were gastrointestinal and corticosteroid-responsive, with no patients requiring second-line treatment [25]. Serial peripheral-blood monitoring showed early recovery of memory T-cell populations, while naïve T-cell recovery remained delayed; thymic recovery and TCR diversity were assessed longitudinally, and CMV-specific CD8+ T cells were detectable by day 28 with functional cytokine responses in seven evaluable CMV-seropositive recipients [25].

This approach was subsequently evaluated in a pooled analysis of three prospective phase II studies involving 138 matched related and unrelated donor recipients. At three years, the cumulative incidence of cGVHD was 7% (moderate 1%, severe 0%), while relapse, NRM and OS were 23%, 8% and 77%, respectively. The cumulative incidence of grade II aGVHD was 71%, largely reflecting mild upper gastrointestinal disease; grade III aGVHD occurred in 4%, and no grade IV disease was observed [49]. The approach was later extended to haploidentical paediatric transplantation. In the Naik et al. study, 3-year OS and EFS were 86.4% and 77.4%, respectively, in patients transplanted in morphological remission. In this cohort, the 1-year cumulative incidence of grade III-IV aGVHD was 29.2%, while the 3-year cumulative incidence of moderate-to-severe cGVHD was 15.6% [50]. These outcomes should be interpreted with caution, as CD45RA depletion was used as part of a more complex transplant strategy that also included CD34 enrichment, NK-cell add-back, short-course immunosuppression and sub-myeloablative conditioning.

Despite this biological appeal, several uncertainties remain. Evidence that retaining memory T cells confers clinically meaningful protection from infection is limited, and the breadth and durability of pathogen-specific immunity across donor types remain undefined [25]. Generalisability is further constrained by regimen heterogeneity: the matched-donor phase II studies used predominantly TBI-based conditioning [49], whereas the paediatric haploidentical study combined CD45RA depletion with CD34+ enrichment, NK-cell add-back, short-course immunosuppression and submyeloablative conditioning [50], leaving the independent contribution of naïve T-cell depletion difficult to isolate. The original manufacturing study achieved reproducible two-step depletion and delivery of a defined memory T-cell dose [48], but reproducibility across centres and processing platforms is untested. Whether preserved memory T cells sustain GVL activity without increasing relapse likewise awaits comparative confirmation [49].

Overall, the available evidence suggests that CD45RA depletion can preserve early memory T-cell recovery while achieving low rates of cGVHD. However, aGVHD is not completely prevented and remains an important limitation. Interpretation of the available evidence is also limited by the lack of randomised comparative trials and differences between studies in donor type, conditioning regimen, T-cell dose and GVHD prophylaxis [25,49,50]. Therefore, although CD45RA depletion provides a more selective approach to graft engineering, its clinical benefit compared with current strategies such as PTCy or other selective depletion approaches remains to be established.

Across CD34^+^ selection, TCRαβ/CD19 depletion and CD45RA^+^ depletion, each strategy trades off some element of immune reconstitution, GVL activity or infection control for reduction in GVHD. This pattern has prompted the need for a complementary approach, leading to exploration of adoptive add-back strategies in which selected lymphocyte cell populations are returned to the graft with the aim of improving outcomes.

## 6. Adoptive Add-Back Strategies

Adoptive add-back strategies are actively evolving and reintroduce defined donor cell populations into the graft, whose alloreactive potential can be controlled or eliminated. In this section, we cover two approaches: (1) regulatory-conventional T-cell (Treg-Tcon) co-infusion and its successor, TREGZI (developed as Orca-T), and (2) suicide gene-modified donor T cells.

The proof of concept for the Treg-Tcon approach came from the first human study in 28 patients who underwent haplo-HSCT for high-risk haematological malignancies. Di Ianni et al. infused CD4^+^CD25^+^ Treg cells 4 days prior to infusion of a megadose of CD34^+^ cells and Tcon cells (defined as CD19^−^ cells) on Day 0. This approach was associated with enhanced immune recovery and reduced GVHD in the absence of post-transplant immunosuppression, with a 1-year DFS of 46.1% [23]. Martelli et al. expanded on this approach in a phase II study involving 43 adults with high-risk acute leukaemia and demonstrated ≥grade II aGVHD in 15% of patients with only a 5% cumulative incidence of relapse; however, the overall DFS at 18-month minimum follow-up was 56%, with NRM presenting as a limiting factor in 40% of the patients overall [24]. Pierini et al. took this platform a step further by combining the Treg-Tcon co-infusion with an age-adapted myeloablative conditioning regimen, with patients up to 50 years of age receiving TBI, and patients greater than 50 (up to 65 years of age) or unfit for TBI receiving total marrow/lymphoid irradiation. This approach was associated with a probability of 75% of moderate/severe cGVHD/relapse-free survival with a cumulative relapse incidence of 4%, and NRM observed in 20% of the patients [51], raising the possibility that tailoring the conditioning regimen to patient age could help in closing the survival gap and contribute to improved outcomes.

Despite this progression, the Treg-Tcon co-infusion approach remained largely confined to specialised single-centre programmes limited by variable Treg yields, non-cryopreserved manufacture and the need for closely coordinated GMP processing and transplantation. Orca-T addressed some of these constraints through centralised manufacture and distribution of a defined matched-donor product. Now licensed as TREGZI, the product comprises purified donor HSPCs and Tregs given on day 0, followed by Tcon cells on day +2 or +3, and is therefore administered as the graft itself rather than as a post-transplant adjunctive cellular therapy [21,22].

On 30 June 2026, the U.S. FDA approved TREGZI for matched-donor HSCT with a myeloablative preparative regimen, for haematopoietic and immunologic reconstitution and to improve cGVHD-free survival in adults with haematological malignancies [21]. The approval was supported by PRECISION-T, a multicentre, open-label, randomised phase III trial in 187 adults with acute leukaemia or MDS receiving G-CSF-mobilised PBSC grafts from HLA-matched donors, randomised to TREGZI plus single-agent tacrolimus (*n* = 93) or an unmanipulated allograft plus tacrolimus/methotrexate (*n* = 94) [21,22]. The primary endpoint, survival free from moderate-to-severe cGVHD, was significantly improved with TREGZI (HR 0.26, 95% CI 0.14–0.47; *p* < 0.001), with 1-year estimates of 78% versus 38.4%. At one year, moderate-to-severe cGVHD was 12.6% versus 44.0%, GRFS was 63.1% versus 30.9%, and NRM was 3.4% versus 13.2% [22]. Relapse at 1 year was numerically higher with TREGZI (21.1% versus 12.7%, not significant), and 1-year OS was 93.9% versus 83.1% (*p* = 0.12) at a median follow-up of 11.4 months. The most common adverse reactions were mucositis, gastrointestinal symptoms, rash and infections; no severe infusion reactions or graft failures occurred, although the prescribing information carries warnings for graft failure, GVHD, infusion reactions, secondary malignancies and transmission of infectious agents [21]. The comparator was tacrolimus/methotrexate rather than PTCy, so the trial establishes superiority over conventional calcineurin-inhibitor prophylaxis but not over contemporary PTCy-based platforms.

Whilst Treg/Tcon platforms are aimed at preventing development of alloreactive GVHD, the suicide gene-modified donor T cells take a different approach by engineering an on-demand safety mechanism to eliminate the T cells if GVHD develops. The proof of concept for this approach came from the TK007 trial, which included T-cell-depleted haplo-HSCT of high-risk leukaemia patients followed by infusions of donor lymphocytes engineered to express herpes simplex thymidine-kinase suicide (HSV-TK) gene. GVHD developed in the patients was controlled by administration of ganciclovir that activated the suicide switch on the HSV-TK cells [53]. However, due to the slow response time of ganciclovir and its role as an antiviral therapeutic product, alternative strategies for mediating suicide gene approaches were explored. This included the use of inducible caspase-9 (iCasp9)-expressing donor T cells, which trigger rapid apoptosis of T cells expressing this construct upon exposure to the bioinert small-molecule dimerising drug, AP1903. In an early-phase study, a single dose of this drug was sufficient to resolve and prevent recurrence of GVHD [54]. Longer-term follow-up showed pathogen-specific immune responses emerging or expanding over subsequent weeks to months after infusion, assessed serially in peripheral blood [55].

This approach was developed into an experimental therapy, BPX-501, and a conference abstract reported a study in paediatric patients suggesting activity in the treatment of steroid-resistant GVHD [56]. Despite these promising preliminary results, this safety switch strategy has not progressed to scalable therapeutic options, likely due to time-consuming and costly manufacturing processes [76]. Unlike the Treg-Tcon approach, which has now been validated in a randomised phase III clinical trial, suicide gene-modified donor T cells have not been tested against a randomised comparator, leaving their benefit relative to established prophylaxis strategies unresolved.

**Figure 2:**
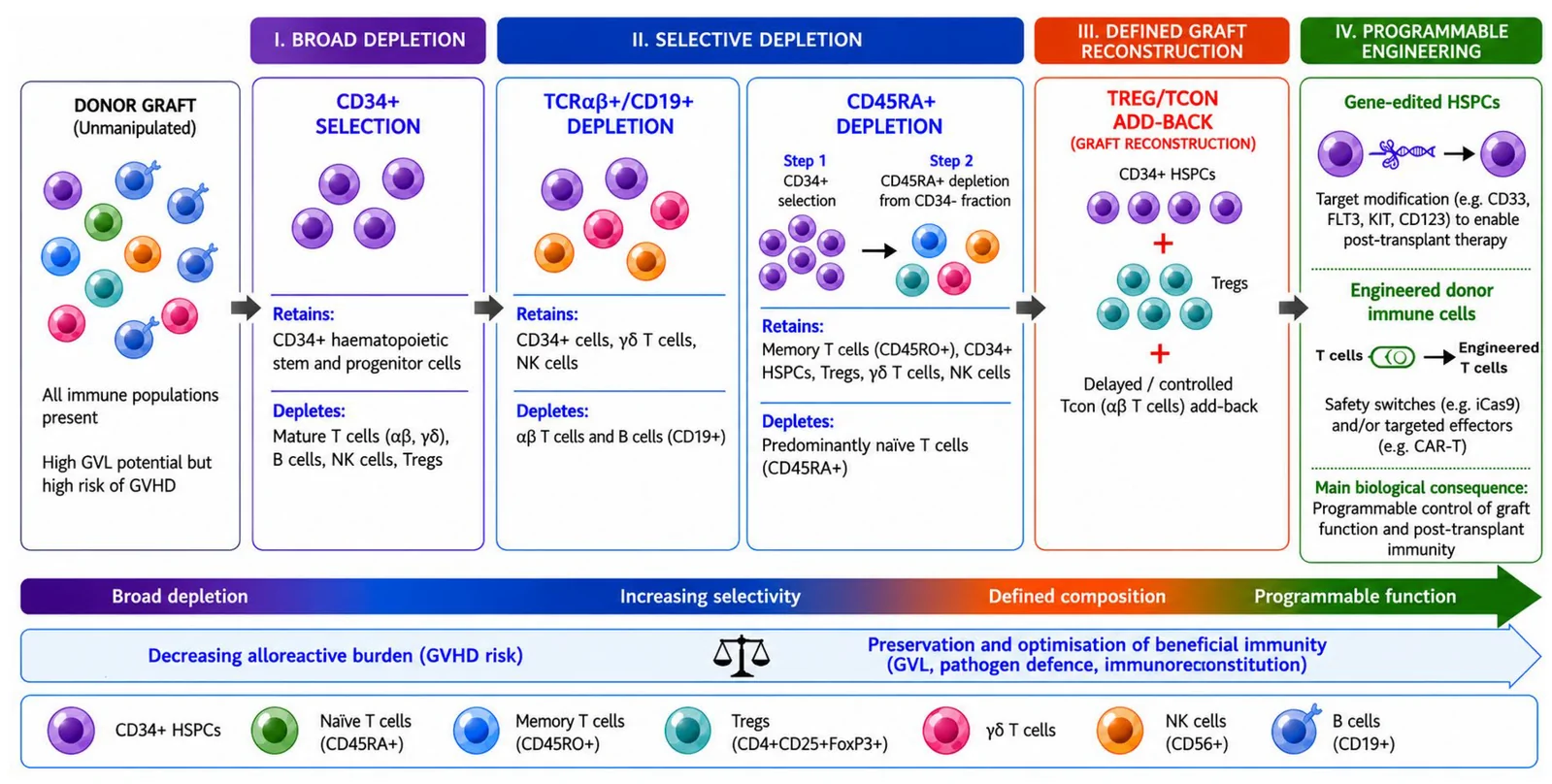
**Evolution of ex vivo graft engineering in allogeneic HSCT: from broad depletion to selective immune manipulation**. Early approaches relied on broad depletion of mature immune-cell populations through CD34^+^ positive selection. Subsequent strategies increased selectivity through αβ^+^/CD19^+^ or CD45RA depletion, aiming to reduce alloreactivity while preserving beneficial immune-cell populations. Defined graft-reconstruction approaches subsequently introduced controlled Treg and Tcon add-back, while emerging gene-editing and cellular-engineering strategies aim to provide programmable control of graft function and post-transplant immunity. The cellular populations shown are schematic and may vary according to the individual manufacturing platform.

The evolution of ex vivo graft engineering from broad depletion towards increasingly selective, defined and programmable graft engineering is summarised in Figure 2.

## 7. Graft Engineering Versus In Vivo T-Cell Depletion

Up until the last 5 years, both ATG and Campath have remained the dominant TCD strategies based on clinical trial evidence mainly compared to no TCD (Supplementary Table S1). Two polyclonal preparations are in use clinically, and they are not interchangeable. Thymoglobulin is produced by immunising rabbits with fresh human thymocytes, whereas anti-T-lymphocyte globulin (ATLG; Grafalon) is raised against a T-lymphoblast cell line. The two differ in dosing and antigen specificity; in MUD transplants, ATLG missed its composite primary endpoint of severe aGVHD or death within 100 days; however, it did reduce the incidence of cGVHD from 42.6% to 12.2% (adjusted HR 0.22, 95% CI 0.11–0.43; *p* < 0.0001) with no difference in relapse [77]. In sibling transplantation, the 2-year cumulative incidence of cGVHD was 32.2% with ATG versus 68.7% without ATG (*p* < 0.001), with no difference in OS [78]. An often-unmeasured metric is freedom from immunosuppression (IS). Walker et al. demonstrated that 38% of patients who received ATG were able to discontinue IS at 24 months compared to 19% who did not (adjusted OR 3.49, 95% CI 1.60–7.60; *p* = 0.0016) [79].

Alemtuzumab is a monoclonal antibody directed at CD52 which depletes both T and B cells and is most widely used in the UK. A recent EBMT registry comparison of ATG and alemtuzumab-based prophylaxis in adults with ALL receiving reduced-intensity conditioning found comparable outcomes between the two agents, with similar 2-year OS (62.7% vs. 62.9%), relapse, NRM, and rates of severe aGVHD and extensive cGVHD [80]. Earlier cohort data showed the same trade-off seen with ATG: alemtuzumab-based nonmyeloablative conditioning reduced TRM and GVHD in unrelated donor transplantation, but at the cost of impaired graft-versus-tumour effect [81]. Both agents also impair immune reconstitution, depleting pan-lymphocyte populations including virus-specific T cells, predisposing to EBV-driven PTLD and CMV reactivation [28,82,83]. Non-selective TCD may therefore incur a relapse penalty through attenuation of the GVL effect [84].

A major development in the past decade has been PTCy, which was developed for haplo-HSCT [17]. PTCy preferentially targets proliferating alloreactive T-cells [85] while preserving Treg subsets through aldehyde dehydrogenase expression [86]. CIBMTR activity data demonstrate the rise in PTCy, which was used in 49% of allogeneic transplants performed in the United States in 2024, compared with 3% in 2014 [87].

Compared with standard methotrexate/tacrolimus, PTCy improved 1-year GRFS from 34.9% to 52.7% in matched or single-mismatched transplants [88]; PTCy with cyclosporin improved 3-year GRFS from 14% to 49% in matched related donor transplantation [89]; and in HOVON-96 the 2-year cumulative incidence of extensive cGVHD was 16% with PTCy versus 48% with standard prophylaxis after nonmyeloablative conditioning [90]. Notably, the one randomised trial where PTCy did not outperform standard prophylaxis administered it as a single agent, without a calcineurin inhibitor [35], whereas each of the positive trials combined PTCy with one. The phase II ACCESS trial [18] tested PTCy-based prophylaxis in 145 recipients of mismatched-unrelated-donor PBSC transplantation. One-year OS was 83.8% after myeloablative conditioning and 78.6% after reduced-intensity/nonmyeloablative conditioning. The 6-month cumulative incidence of grade III-IV aGVHD was 8.0% and 10.0%, respectively, and the 1-year cumulative incidence of moderate/severe cGVHD was 10.3% and 8.6% [18].

Until recently, comparisons between PTCy and ATG/Campath were largely retrospective. Registry analyses reported better survival with PTCy than with ATG in mismatched unrelated donor transplantation [14]. A meta-analysis of nine retrospective comparisons in haplo-HSCT reported the same finding [91]. In matched related donors, however, only relapse differed between the two approaches, but survival did not [92]. Randomised evidence has not borne this out. A small open-label phase II trial [93] found no significant difference in OS, RFS or GVHD between ATG and PTCy in fully matched transplants. Results from the GRAPPA trial, recently presented at the EHA 2026 congress [94], found that PTCy reduced both acute and chronic GVHD more effectively than ATG in MUD transplants, yet failed to demonstrate non-inferiority for OS [94]. The PTCy arm also had higher NRM (2-year NRM 13% vs. 7%; *p* = 0.02), driven by a higher infection rate. A three-arm randomised trial in haplo-HSCT similarly found no survival difference between PTCy, ATG, or the two combined [95]. Neither agent has therefore been shown superior, and the recurring observation across this literature is that better GVHD control does not reliably translate into an OS benefit.

Ex vivo manipulation may represent an important step in the separation of GVL and GVHD. Pierini et al. noted a powerful GVL effect using a defined Treg:Tcon infusion combined with CD34^+^ stem cells [51] with a low cumulative relapse rate of 4% in high-risk AML patients. They postulate this may be due to differential homing: Tcons trafficked to the bone marrow, where they could exert GVL activity, whereas Tregs homed to tissues instead, thereby preventing GVHD. In the 50-patient cohort, grade ≥II aGVHD had a cumulative incidence of 33% (15 patients; median onset 41 days); moderate/severe cGVHD occurred in only one patient [51]. Orca-T builds on this through a defined ratio of all three populations of haematopoietic progenitor cells, Tregs and Tcons [22]. Both programmes reported encouraging immune-reconstitution and infection outcomes, although these findings arose from separate studies and should not be interpreted as evidence of comparative superiority.

Whether precision engineering can reduce or remove the need for post-transplant immunosuppression remains to be established. In a randomised 24-patient comparison, grade II-IV aGVHD occurred in 7/12 patients (58%) receiving the Treg-engineered graft without prophylaxis versus 1/12 (8%) single-agent tacrolimus; moderate/severe cGVHD occurred in 28% versus 0%, respectively, and the authors concluded that tacrolimus should be retained [96]. By contrast, in haplo-HSCT, Di Ianni et al. and Martelli et al. delivered their engineered graft with no IS and no serotherapy, reporting grade II or higher aGVHD in 15% of patients and a relapse incidence of 5% [23,24]. PRECISION-T, which retained a calcineurin inhibitor, halved the regimen to single-agent tacrolimus [22]. Freedom from IS is already used as a trial endpoint in its own right [79], and immunosuppression-free protocols are in prospective testing (NCT04678401; NCT07366801). An immunosuppression-free transplant could offer clinical advantages by reducing exposure to immunosuppressive therapy, although this remains to be demonstrated prospectively.

Few studies have directly compared ex vivo techniques to in vivo TCD. The EBMT Inborn Errors Working Party compared TCRαβ/CD19 depletion with PTCy in 306 children receiving HLA-mismatched transplants for inborn errors of immunity. In this registry cohort (TCRαβ/CD19 *n* = 167; PTCy *n* = 139), the cumulative incidence of severe aGVHD was 6% versus 15%, respectively, while 3-year OS was 78% versus 66% in univariate analysis [44]. EFS was similar (58% vs. 57%), and on multivariable analysis the only independent predictors of survival were pre-transplant morbidity and the conditioning backbone. Cyclophosphamide was associated with more veno-occlusive disease, acute kidney injury and pulmonary complications, while ex vivo depletion was associated with more adenoviraemia, primary graft failure and second transplants. BMT CTN 1301, discussed above, reported significantly increased TRM, which nearly trebled (HR 2.76, 95% CI 1.26–6.06) [35]. Together, these studies illustrate the trade-offs that remain with ex vivo depletion strategies: reducing alloreactive T-cell burden may improve GVHD control, but can also alter immune recovery and infection risk, with the balance differing substantially between platforms.

PRECISION-T [22], the TREGZI (Orca-T) registration study, was a prospective phase III trial comparing the defined-composition graft with standard methotrexate/CNI prophylaxis and no added in vivo TCD. No randomised trial has compared a precision-engineered graft with PTCy; however, two non-randomised comparisons have reported results favouring engineering. A multicentre cohort of 145 adults given TCRαβ-depleted grafts with memory T-cell add-back achieved better GRFS than a propensity-matched cyclophosphamide comparator [57]. A comparison of phase Ib Orca-T recipients against CIBMTR registry controls reported 3-year survival of 83% vs. 66% (HR 0.41), which held under propensity matching and multivariable adjustment (HR 0.38, 95% CI 0.21–0.71), and was driven by NRM of 3.1% vs. 10%, with no difference in relapse [52]. Both comparisons are susceptible to selection bias and differences in era, conditioning and supportive care.

Comparison across these two literatures requires caution, because they are not drawn from the same populations. The engineered-graft evidence is weighted towards haploidentical transplantation in children for TCRαβ/CD19 depletion [39,44,45] and towards matched-donor transplantation in adults after myeloablative conditioning for CD45RA^+^ depletion and Orca-T [22,49], whereas the PTCy evidence base spans all donor types, both graft sources and the full range of conditioning intensity [17,18,87,88,89,90]. Nor is PTCy a uniform comparator, since the regimens cited above differ in their accompanying immunosuppression. Graft source can confound even randomised comparisons: in BMT CTN 1301 the CD34-selected arm received PBSC whereas the PTCy and control arms received bone marrow [35]. Methodological drift compounds this, as GVHD grading systems and definitions have changed over time and studies differ in their competing-risk methods, landmark definitions and follow-up duration. Favourable outcomes reported from protocol-defined cohorts at a small number of highly experienced programmes may therefore not be reproducible in broader practice [24,39,49,51].

Low chronic GVHD does not by itself establish superiority over in vivo TCD; that requires a broader set of outcomes including OS, RFS, GRFS, relapse, NRM, serious infection and immune reconstitution, alongside quality of life, duration of systemic immunosuppression, hospital utilisation, late effects, feasibility and cost [79,97]. On these accounts, the randomised evidence remains thin. CD34^+^ selection reduced chronic GVHD but increased TRM and lowered OS against both PTCy and tacrolimus/methotrexate [35], and TREGZI improved chronic GVHD-free survival and reduced NRM against tacrolimus/methotrexate but has not been tested against PTCy [22]. No randomised trial has compared TCRαβ/CD19 depletion, CD45RA^+^ depletion or a Treg/Tcon-defined graft with optimised PTCy-based prophylaxis, so claims of superiority over PTCy rest on non-randomised, registry or historical comparisons [44,45,52,57] and should be regarded as hypothesis-generating.

## 8. Future Directions

The approval of an engineered graft is an important milestone; however, it will be some time before randomised evidence comparing these approaches head-to-head becomes available. That gap is not confined to graft engineering. Both ATG and PTCy were adopted on the strength of trials comparing them against prophylaxis containing no TCD at all, rather than against one another [77,78,79,88,89]. Adequately powered head-to-head comparisons have only recently begun. The GRAPPA trial, randomising 640 patients undergoing unrelated donor transplantation to PTCy or ATG with OS as a co-primary endpoint (NCT05153226) was reported at EHA 2026, showing better GVHD control with PTCy but failure to demonstrate non-inferiority for OS; full peer-reviewed results are awaited; the Methods of T-Cell Depletion (MoTD) trial is comparing PTCy + calcineurin inhibitor (CNI) or PTCy + sirolimus to ATG-based GVHD prophylaxis [98]. A direct randomised comparison of an engineered graft against in vivo depletion is a further step, and such trials recruit slowly: Orca-Q, testing a precision-engineered graft in the mismatched setting, has been open since 2019 (NCT03802695).

Transplant populations are heterogeneous, eligible patients are few, and precision engineering adds a further bottleneck to large transplant trials. Methods that reduce the number of patients required may therefore matter as much as methods that improve the graft itself. Firstly, external and synthetic control arms, derived from registry and real-world data, are being formally evaluated in oncology, with their survival estimates tested for reproducibility against completed randomised trials [99]. Secondly, artificial intelligence is being applied to trial design, patient eligibility and endpoint selection [100]. Applied carefully, both approaches could make head-to-head comparisons of these technologies more feasible.

Gene editing of donated haematopoietic stem cells to enable post-transplant maintenance is an early-phase area of graft engineering. In a phase I/II trial in high-risk AML, the surface marker CD33 was deleted from donor stem cells using CRISPR-Cas9. CD33 is expressed both on normal myeloid cells and leukaemic blasts; hence CD33-directed therapy can also damage normal donor-derived myeloid cells. Deleting CD33 from the graft is intended to protect normal haematopoiesis while allowing post-transplant treatment to target residual CD33-positive leukaemic cells. All 30 patients engrafted and CD33-negative haematopoiesis persisted at close to 100% through repeated cycles of gemtuzumab ozogamicin, the CD33-directed antibody-drug conjugate. No safety signals specific to gene editing were reported, although there was no randomised comparator and the trial was terminated early for financial rather than clinical reasons [61]; a second CD33-editing programme is planned in Europe (NCT05662904). This same principle is also being applied through base editing of the small region of FLT3, KIT or CD123 recognised by a therapeutic antibody, leaving the protein functional but the graft resistant, extending the approach to targets that cannot simply be deleted [101].

A related but distinct area is donor-derived engineered immune-cell therapy, including products administered after allo-HSCT or developed independently of the transplant graft. HA-1-specific TCR-engineered donor T cells and donor-derived anti-CD33 CAR T cells have shown early clinical activity after allo-HSCT [102,103], and TCR-disrupted donor-derived CAR-T products, including base-edited CAR7 cells, illustrate the potential to reduce alloreactivity while retaining antileukaemic function [104]. A trial combining a Treg-engineered graft with CD19/CD22 CAR T cells has completed accrual (NCT05507827). These are adoptive cellular therapies rather than modifications of the primary haematopoietic graft and should not be regarded as established graft-engineering platforms. These approaches are not cheap, and no peer-reviewed analysis has yet compared the cost-effectiveness of ex vivo graft engineering with PTCy. PTCy is an inexpensive generic drug requiring no specialised infrastructure, no additional donor work-up and no cell processing, making it a competing standard that is hard to beat on cost alone. However, if graft engineering reduces infectious complications and cGVHD, downstream savings may offset its high manufacturing costs. A conference abstract from PRECISION-T reported that TREGZI recipients required only single-agent tacrolimus rather than tacrolimus and methotrexate, and had fewer grade III infections at one year (8.4% vs. 16.1%), with a single case of CMV reactivation across the entire treatment arm; they were also rehospitalised for adverse events less often (27.3% vs. 45.7%), and required intensive care less often (1.1% vs. 4.3%) [97]. A manufacturer-sponsored cost-effectiveness analysis proposes that savings accrued over a transplant recipient’s lifetime may offset the upfront costs [105]; however, this is currently reported only in abstract form and remains to be confirmed in larger numbers.

A further consideration is distribution, as graft engineering aims to improve outcomes even in mismatched allografts—a stem cell source that remains vital for ethnic minorities. If the engineering that makes those transplants safer is available only in a handful of specialist centres, the field risks reproducing the very inequity it set out to solve. Within a single prospective trial, six independent sites each manufactured grafts to specification serving the twelve participating centres using the Miltenyi platform [20], and the same automated platform has supported point-of-care CAR T-cell manufacture in middle-income countries [106,107]. Semi-automation lowers the expertise threshold while maintaining manufacturing standards, and point-of-care manufacture has been proposed explicitly as a route to equity of access [108].

## 9. Clinical Positioning

Where graft engineering will sit within modern transplant programmes remains uncertain. The clearest case is selected paediatric haploidentical transplantation within programmes that already have established graft-processing, quality-control and infection-support infrastructure, where TCRαβ/CD19 depletion has the largest clinical experience [39,72]. A second setting is where reducing the intensity or duration of post-transplant pharmacological immunosuppression is a specific clinical objective, although this potential benefit is platform-dependent and must be weighted against the risks of delayed immune recovery, infection, graft failure and relapse. In adults undergoing myeloablative matched-donor transplantation for haematological malignancy in the United States, TREGZI (Orca-T) is now a licensed graft-engineering option; however, its pivotal trial compared the product with tacrolimus/methotrexate rather than with optimised PTCy [22], and its comparative effectiveness against contemporary PTCy-based prophylaxis therefore remains uncertain.

Outside established or approved settings, these approaches are most appropriately used within protocol-driven programmes with validated manufacturing, release testing and supportive-care procedures, or in research studies testing specific hypotheses about graft immune-cell composition. Choice of platform should therefore reflect donor source, disease, conditioning regimen, patient age, centre expertise and the clinical priority being addressed, rather than an assumption that one engineering strategy is universally superior. PTCy remains a broadly accessible, inexpensive and operationally simple reference strategy across matched and mismatched donor settings. No selective graft-engineering platform has yet demonstrated definitive superiority over optimised PTCy in a head-to-head randomised trial.

## 10. Conclusions

The last 20 years have seen remarkable progress within the field of stem cell transplantation. The question posed at the outset of this review, how to separate GVHD from GVL, remains unresolved; however, the engineering approaches now available come closer to answering it than anything that preceded them. Selective graft engineering can reduce chronic GVHD and non-relapse mortality in defined settings, and TREGZI is now U.S. FDA-approved for matched-donor HSCT with myeloablative conditioning in adults with haematological malignancies [21]. Whether graft engineering offers a survival advantage over modern in vivo prophylaxis, including PTCy, remains unresolved and requires direct randomised comparisons. These technologies require infrastructure that most centres do not have and compete with an inexpensive generic drug that requires none, so in health systems under financial pressure, cost and capacity may determine adoption as much as efficacy. The key unanswered question is therefore not simply whether selective graft engineering can lower GVHD in selected cohorts, but whether it improves patient-centred outcomes compared with optimised contemporary in vivo prophylaxis in defined donor, disease and transplant settings.

## Figures and Tables

**Table 1:** **Platform-level summary of selective graft-engineering and related strategies in allogeneic HSCT.** Platforms span different donor, disease, age and conditioning settings. Evidence levels are stated explicitly as randomised controlled trial (RCT), non-randomised comparative, prospective single-arm, retrospective or registry evidence. Detailed trial-level outcomes are given in Supplementary Table S1. Post-transplant cellular add-back is included for clinical context but is distinct from ex vivo manipulation of the haematopoietic graft before infusion. Cord-blood expansion is excluded because it primarily alters progenitor-cell dose rather than immune composition. The ‘Principal cellular manipulation’ column identifies the immune-cell populations that are enriched, removed, retained or added back for each platform.

Platform	Principal Cellular Manipulation	Typical Transplant Setting	Graft Source	Post-Transplant GVHD Prophylaxis	Evidence Level
CD34^+^ positive selection	Enriched: CD34^+^ HSPC (megadose in haplo-HSCT) Removed: essentially all mature T, B and NK cells	Haploidentical [13,36] HLA-matched related/unrelated [35,37]	G-CSF PBSC	None (CNI-free) [13,35,36,37] ATG in haplo conditioning [36]	Phase III RCT: BMT CTN 1301 [35] Non-randomised comparative: BMT CTN 0303 [37] Prospective single-arm [13,36]
TCRαβ/CD19 depletion	Removed: TCRαβ^+^ T cells and CD19^+^ B cells Retained: CD34^+^ HSPC, γδ T cells, NK cells, monocytes [38]	Predominantly haploidentical [20,39] Also matched related and unrelated donors [40,41,42]	G-CSF PBSC	Often none post-transplant [39,43] ATG in conditioning in several protocols [20,42]	Prospective single-arm phase I/II (paediatric [39]; adult + paediatric [20] Retrospective comparative vs. PTCy: paediatric IEI, registry [44] and single centre [45] Non-randomised single-centre adult comparison vs. T-replete + ATG [42] No RCT
CD3/CD19 depletion	Removed: all CD3^+^ T cells and CD19^+^ B cells Retained: CD34^+^ HSPC, NK cells	Haploidentical, adults [46,47]	G-CSF PBSC	OKT-3 [47]	Phase II multicentre [47]
CD45RA+ (naïve T-cell) depletion	Two-step: CD34^+^ selection, then CD45RA^+^ depletion of the CD34^−^ fraction [25,48] Removed: naïve T cells Retained/added back: memory T cells at a defined dose	HLA-matched related/unrelated [25,49] Haploidentical, paediatric, with NK add-back [50]	G-CSF PBSC	Tacrolimus alone [25] Tacrolimus ± methotrexate or MMF [49] Short-course sirolimus or MMF in paediatric haploidentical protocol [50]	Prospective single-arm phase I/II [25] Pooled three phase II trials [49] Phase II [50]
Defined Treg/Tcon graft (Perugia)	Enriched: CD34^+^ megadose Added back: donor CD4^+^CD25^+^ Treg (day −4), then Tcon (day 0) [23]	Haploidentical, adults [23,24,51]	G-CSF PBSC; fresh, non-cryopreserved manufacture	None [23,24,51]	Prospective single-centre phase I–II [23,24,51]
TREGZI (Orca-T)	Defined composition: HSPC + Treg (day 0) + Tcon (day +2/+3) Central GMP manufacture [22]	HLA-matched donors, adults, MAC [22]	G-CSF PBSC	Single-agent tacrolimus [22]	Phase III RCT vs. Tac/MTX (PRECISION-T) [22]; U.S. FDA approved 2026 [21] Phase Ib vs. CIBMTR registry controls [52]
Post-transplant cellular add-back (related approach; distinct from primary graft engineering)	Added after a T-cell-depleted graft: suicide-gene DLI (HSV-TK [53]; iCasp9 [54,55]; BPX-501 [56]); CD45RA-depleted memory DLI [57,58]; CD8-depleted DLI [59]; photodepleted DLI (ATIR101) [60]	Mostly haploidentical	CD34-selected or αβ-depleted PBSC + DLI	Variable; none with ATIR101 [60]	Early-phase studies [53,54] Phase II with observational comparator [60] RCTs [58,59] Prospective multicentre single-arm study with propensity-matched PTCy comparison [57]
Gene-edited HSPC graft (CD33-deleted; trem-cel)	CRISPR–Cas9 deletion of CD33 in donor CD34^+^ cells, allowing post-transplant gemtuzumab ozogamicin [61]	Adults with high-risk CD33+ AML/MDS; 8/8 HLA-matched related or unrelated donors [61]	G-CSF/plerixafor-mobilised PBSC; CD34+ selected and CRISPR–Cas9-edited HSPCs [61]	No post-transplant GVHD prophylaxis; ATG included in conditioning [61]	Phase I/IIa, multicentre, open-label [61]

Abbreviations: AML, acute myeloid leukaemia; ATG, anti-thymocyte globulin; BM, bone marrow; CNI, calcineurin inhibitor; DLI, donor lymphocyte infusion; G-CSF, granulocyte colony-stimulating factor; GMP, good manufacturing practice; HLA, human leukocyte antigen; HSPC, haematopoietic stem and progenitor cells; IEI, inborn errors of immunity; MAC, myeloablative conditioning; MDS, myelodysplastic syndrome; MMF, mycophenolate mofetil; PTCy, post-transplant cyclophosphamide; RCT, randomised controlled trial; Tac/MTX, tacrolimus/methotrexate; Tcon, conventional T cells; Treg, regulatory T cells.

## Data Availability

No new data were created or analysed in this study. Data sharing is not applicable to this article.

## Supplementary Materials

The following supporting information can be downloaded at: https://www.mdpi.com/article/10.3390/jcm15218407/s1, Table S1: Selected clinical studies of graft engineering and comparator strategies in allogeneic HSCT. References [109,110,111,112] are cited exclusively in Supplementary Table S1.

## Abbreviations

aGVHD	Acute graft-versus-host disease
ALL	Acute lymphoblastic leukaemia
allo-HSCT	Allogeneic haematopoietic stem cell transplantation
AML	Acute myeloid leukaemia
ATG	Anti-thymocyte globulin
ATLG	Anti-T-lymphocyte globulin
BMT CTN	Blood and Marrow Transplant Clinical Trials Network
CAR	Chimeric antigen receptor
CIBMTR	Center for International Blood and Marrow Transplant Research
CMV	Cytomegalovirus
CNI	Calcineurin inhibitor
CR	Complete remission
CRFS	Chronic GVHD/relapse-free survival
CRISPR-Cas9	Clustered regularly interspaced short palindromic repeats/CRISPR-associated protein 9
cGVHD	Chronic graft-versus-host disease
DFS	Disease-free survival
DLI	Donor lymphocyte infusion
EBV	Epstein–Barr virus
EFS	Event-free survival
FDA	Food and Drug Administration
G-CSF	Granulocyte colony-stimulating factor
GMP	Good manufacturing practice
GRFS	GVHD-free, relapse-free survival
GVHD	Graft-versus-host disease
GVL	Graft-versus-leukaemia
HLA	Human leukocyte antigen
HSPC	Haematopoietic stem and progenitor cell
HSCT	Haematopoietic stem cell transplantation
HSV-TK	Herpes simplex virus thymidine kinase
iCasp9	Inducible caspase 9
IS	Immunosuppression
KIR	Killer-cell immunoglobulin-like receptor
MAC	Myeloablative conditioning
MDS	Myelodysplastic syndrome
MHC	Major histocompatibility complex
miHA	Minor Histocompatibility antigen
MMF	Mycophenolate mofetil
MMUD	Mismatched unrelated donor
MRD	Measurable residual disease
MTX	Methotrexate
MUD	Matched unrelated donor
NK	Natural killer
NMA	Non-myeloablative conditioning
NRM	Non-relapse mortality
OS	Overall survival
PBSC	Peripheral blood stem cells
PFS	Progression-free survival
PTCy	Post-transplant cyclophosphamide
PTLD	Post-transplant lymphoproliferative disorder
RIC	Reduced-intensity conditioning
RFS	Relapse-free survival
TBI	Total body irradiation
TCD	T-cell depletion
Tcon	Conventional T cell
TCR	T-cell receptor
TCRαβ	αβ T-cell receptor
Treg	Regulatory T cell
TRM	Transplant related mortality
