# Supplementary material for: From Pan-T-Cell Depletion to Selective Graft Engineering: Progress, Limitations, and Open Questions
Source: J Clin Med. 2026 Oct 28;15(21):8407. doi: 10.3390/jcm15218407 (PMC13764376; doi:10.3390/jcm15218407)
Supplement: Supplementary file 1 [file jcm-15-08407-s001.zip › jcm-4561838-supplementary.pdf]

Supplementary Table S1. Selected clinical studies of graft engineering and comparator strategies in allogeneic HSCT

Study populations, donor types, graft sources, conditioning regimens, GVHD prophylaxis, endpoint definitions and duration of follow-up vary substantially between studies; outcome estimates should therefore not be compared directly across rows. Post-transplant cellular add-back strategies are included for clinical context but are distinct from ex vivo manipulation of the primary haematopoietic graft. Outcomes are reported according to the definitions used in the cited studies. NR, not reported.

Trial (ref)	Design, n	Population / donor	Intervention	GvHD	Relapse & NRM	Survival	Key limitations / interpretive considerations
**A. In vivo T-cell depletion**							
**A1. Antithymocyte globulin and anti-T-lymphocyte globulin**							
**Finke 2009** [1]	Phase III RCT, open-label<br>n = 201 (ATLG 103, control 98)	Adults, haematological malignancy; matched unrelated; myeloablative; CsA + MTX ± ATLG	ATLG (ATG-Fresenius, anti-Jurkat) vs standard	CI aGvHD II–IV 33.0% vs 51.0% (HR 0.56, 0.36–0.87; P = 0.011)<br>CI aGvHD III–IV 11.7% vs 24.5% (HR 0.50, 0.25–1.01; P = 0.054)<br>2-year extensive cGvHD 12.2% vs 42.6% (HR 0.22, 0.11–0.43; P< 0.0001)	No difference in relapse or Day 100 NRM (ATG-F n=11, 10.7%; control n=13,13.3%)<br><br>2-year NRM ATG 19.6% (95% CI 13.2-29) vs control 28.9% (95% CI 20.8-40.1) adj HR0.68 (95% CI 0.38-1.22; P = 0.20)<br><br>2-year Relapse ATG-F 28.9% (95% Ci 21.2-39.3) vs control 23.6% (95% CI 16.5-33.7) adj HR 1.18, (95% CI:0.69-2.03; P = 0.55)	2-year OS ATG-F 59.2% (95% CI 49.5-68.9) vs 51.9% (95% CI 41.3-62.5) adj HR 0.856 (95% CI 0.57–1.30)	Primary endpoint NOT met (severe aGvHD or death by day 100: 21.4% vs 33.7%, P = 0.13). Large chronic GvHD benefit, no survival benefit.
**Soiffer 2017**<br>[2]	Phase III RCT, double-blind, placebo-controlled<br>n = 254	Adults 18–65, acute leukaemia or MDS; matched unrelated; myeloablative	ATLG (Neovii) 20mg/kg D -3,-2,-1 vs placebo added to tacolimus and IV Methotrexate 15mg/m2 on day 1 then 10mg/m2 days 3, 6, 11	aGvHD II–IV 23% vs 40% (*P* = .004)<br>Moderate–severe cGvHD 12% vs 33% (*P* < .001)	2-year CI NRM 21% (95% CI 14-29%) in ATLG vs 13.5% (95% CI 8-20%) in placebo P = 0.53<br><br>2-year relapse 32% (95% CI 23-41) ATLG vs 21% (95% CI 14-29) placebo P = 0.1)	2-year mod–severe cGvHD-free survival 48% vs 44% (P = 0.47)<br>2-year PFS 47% vs 65% (P = 0.04)<br>2-year OS 59% vs 74% (P =0.034)<br>Multivariable: PFS HR 1.55 (1.05–2.28); OS HR 1.74 (1.12–2.71), P = 0.01	Primary endpoint NOT met:<br><br>The only double-blind trial, and the only one showing net harm. GvHD substantially reduced yet survival significantly worse.

Trial (ref)	Design, n	Population / donor	Intervention	GvHD	Relapse & NRM	Survival	Key limitations / interpretive considerations
**Kröger 2016**[3]	Phase III RCT, open-label<br>n = 168	Adults, acute leukaemia; HLA-identical sibling; PBSC; myeloablative	Anti-human T lymphocyte globulin (ATG)<br><br>10mg/kg day -3,-2,-1 along side cyclosporine and Methotrexate 15mg/m2 on day 1 then 10mg/m2 days 3, 6, 11	GradeII-IV aGVHD 10.8% ATG vs 18.1% control: P = 0.13<br><br>2-year cGvHD 32.2% vs 68.7% (P < .001)<br>No difference in acute GvHD	2-year NRM 14% (95% CI 8-24) in ATG vs 12% (95% CI 6.1-22.4) Non -ATG P = 0.60<br><br>2-year Relapse 32.2% (95% CI 23.4-44.2) ATG vs 25.5% (95% CI 16.3-44.2) non ATG P = 0.17)	2-year RFS 59.4% vs 64.6% (*P* = 0.21)<br>2-year OS 74.1% vs 77.9% (*P* = 0.46)<br>2-year cGvHD-free RFS 36.6% vs 16.8% (P = 0.005)	Extends serotherapy to sibling donors. Large chronic GvHD reduction, no survival benefit.
**Walker 2020**<br>[4]	Phase III RCT, open-label<br>n = 203 (196 evaluable)	Adults 16–70, haematological malignancy; unrelated (matched or 1-locus mismatch); MAC or RIC	Thymoglobulin (rabbit ATG) 4.5 mg/kg	24-mo cGvHD 26.3% vs 41.3% (P = 0.032)<br>Lee-scale symptom burden and depression scores both better with ATG	24-mo relapse 16.3% vs 17.5% (P = 0.73)<br>24-mo NRM 21.2% vs 31.3% (P = 0.15, lower with ATG)	Primary endpoint met: free from immunosuppression at 24 mo 38% vs 19% (OR 3.49, 1.60–7.60; P = 0.0016)<br>24-mo OS 70.6% vs 53.3% (HR 0.56, 0.35–0.90; P = 0.017)	The only trial showing a survival benefit. Serious adverse events fewer with ATG (38% vs 51%). Endpoint was treatment-independence, not GvHD incidence.
**A2. Alemtuzumab**							
**Kottaridis 2000**<br>[5]	Single-arm phase II<br>n = 44	Adults (median 41 y), haematological malignancy; 36 sibling PBSC, 8 MUD marrow; nonmyeloablative (Campath/fludarabine/melphalan)	Alemtuzumab 20 mg/day, days −8 to −4	No grade III–IV acute GvHD<br>Grade II acute GvHD in 2 patients<br>Chronic GvHD in 1 patient	NRM 11%<br>7 relapsed/progressed	33/44 alive in remission at median follow-up 9 months	Not randomised, small, short follow-up (median 9 months). Striking GvHD control, but no comparator and no long-term data.
**Chakraverty 2002**<br>[6]	Single arm<br><br>*N=47*	Adults with no matched sibling	Alemtuzumab	Grade II-IV aGvHD 10 (21.3%)	D100 NRM 14.9% (95% CI 4.7-25.1)<br><br>High incidence of CMV infection; delayed immune reconstitution	1-year OS 75.5% (95% CI, 62.8%-88.2%)<br><br>1-year progression-free 61.5% (95% CI, 46.1%-76.8%)	The cost side of alemtuzumab.
**A3. Post-transplant cyclophosphamide**							
**Luznik 2008**<br>[7]	Single-arm phase I/II<br>n = 68	Advanced haematological malignancy; haploidentical; nonmyeloablative; T-replete bone marrow	PTCy 50 mg/kg day +3 (n = 28) or days +3 and +4 (n = 40)	Day-200 aGvHD II–IV 34%, III–IV 6%<br>Trend to less extensive cGvHD with 2 doses (P = 0.05)	1-year NRM 15%<br>1-year relapse 51%<br>Graft failure 9/66 (13%)	Actuarial OS at 1-year 46% and 2-year 36%<br><br>Actuarial EFS at 1-year 34% and 2-year 26%	The foundational PTCy study. High relapse (51%) in an advanced-disease population; established acceptable graft failure and GvHD rates

Trial (ref)	Design, n	Population / donor	Intervention	GvHD	Relapse & NRM	Survival	Key limitations / interpretive considerations
**BMT CTN 1703**<br>Bolaños-Meade 2023<br>[8]	Phase III RCT<br>n = 431 (214 vs 217)	Adults, haematological cancers; matched related, or matched / 7-8 mismatched unrelated; reduced-intensity	PTCy + tacrolimus + MMF vs tacrolimus + methotrexate	Less severe acute and chronic GvHD; higher rate of immunosuppression-free survival<br><br>12mth cGVHD 21.9% (95% CI 16.4-27.9(PTCy vs 35.1(95% CI 28.7-41.6) control	CI 1-year relapse 20.8% (95% CI, 15.5-26.7) PTCy vs 20.2% (95% CI 15-25.9) HR 0.98 (95% Ci 0.64-1.52)	Primary endpoint met: 1-year GRFS 52.7% vs 34.9%, HR 0.64 (0.49–0.83), P = .001	The pivotal PTCy trial RIC only — does not establish PTCy in myeloablative transplant.
**ALLG BM12 CAST**<br>Curtis 2025<br>[9]	Phase III RCT<br>n = 134 (66 vs 68)	Adults, blood cancers; matched related donor; myeloablative or RIC; PBSC	PTCy + cyclosporin vs cyclosporin + methotrexate	3-mo aGvHD III–IV 3% (1–10) vs 10% (4–19)	Included in composite endpoint	Primary endpoint met: median GRFS 26.2 vs 6.4 months (P < .001)<br>3-year GRFS 49% (36–61) vs 14% (6–25), HR 0.42 (0.27–0.66)<br>2-year OS 83% vs 71% (HR 0.59, 0.29–1.19, NS)	Answers the matched-sibling question, and covers myeloablative conditioning. Largest effect size in the PTCy literature. Serious adverse events similar at 100 days.
**HOVON-96**<br>Broers 2022<br>[10]	Phase III RCT<br>n = 151 (52 vs 99)	High-risk haematological malignancy; matched related or ≥8/8 unrelated; nonmyeloablative; PBSC	PTCy + short-course cyclosporin vs cyclosporin + mycophenolic acid	6-mo aGvHD II–IV 30% vs 48% (HR 0.48, 0.29–0.82; *P* = .007)<br>2-year extensive cGvHD 16% vs 48% (HR 0.36, 0.21–0.64; *P* < .001)	Relapse not significantly different	1-year GRFS 45% (35–55) vs 21% (11–32), P < .001<br>PFS and OS not significantly different (median follow-up 56.4 mo)	Consistent with CAST and 1703. GvHD benefit large; survival unchanged.
**ACCESS**<br>Al Malki 2025 [11]	Phase II, non-randomised, 2 strata<br>n = 145	Adults; mismatched unrelated donor, PBSC; 59% from under-represented racial/ethnic groups	PTCy + tacrolimus + MMF	6-mo aGvHD III–IV: 8% (MAC), 10% (RIC/NMA)<br>1-year mod–severe cGvHD: 10.3% (MAC), 8.6% (RIC/NMA)	Not separately reported	1-year OS 83.8% (MAC), 78.6% (RIC/NMA)<br>Similar OS in patients matched at <7/8	Single-arm; no comparator. Supports donor *availability*, not a claim that mismatch no longer matters (cf. Arrieta-Bolaños 2024).
**A4. Head-to-head comparisons between in vivo agents**							
**GRAPPA**<br>DKMS 21-01<br>NCT05153226<br>*[12]* - available as an abstract only.	Phase III RCT<br>n = 640<br><br>PTCy n=383 vs ATLG n=257	Adults with non-favourable-risk AML, MDS or MDS/MPN; matched unrelated donor; PBSCT	PTCy vs ATLG (Grafalon)<br>Co-primary endpoints: overall survival and GRFS				PTCy suppressed acute and chronic GvHD more effectively than ATLG, including moderate-to-severe disease, however did not demonstrate non-inferiority for overall survival. Reported as supporting ATLG as standard of care in matched unrelated donor transplantation.

Trial (ref)	Design, n	Population / donor	Intervention	GvHD	Relapse & NRM	Survival	Key limitations / interpretive considerations
**Penack 2025** [13]	EBMT registry, retrospective, multivariable-adjusted<br>n = 5,209 (rATG 4,140 vs PTCy 1,069)	Adults, haematological malignancy; matched related donor; transplanted 2017–2021	PTCy vs rabbit ATG	aGvHD II-IV ATG vs PTCy 19.5% (18.2–20.7) vs 21.4% (18.9–24) HR 1.10 (0.88 to 1.37) P = 0.39<br><br>Any grade cGVHD ATG vs PTCy 28.9% (27.4–30.5) vs 34.4% (31.2–37.6) HR 1.01 (0.83 to 1.23 P = 0.90	NRM ATG 12.5% (11.4–13.6) vs PTCy13.1% (10.9–15.4) HR1.22 (0.95 to 1.57) 0.12<br><br>Relapse ATG vs PTCy 32.8% (31.2–34.4) vs 26.2% (23.2–29.2) HR 0.78 (0.66 to 0.92) P = 0.003	OS ATG vs PTCy 64.8% (63.1–66.4) vs 68.5% (65.2–71.6) HR 0.99 (0.85 to 1.16) = 0.92	Lower relapse with PTCy: 26.2% vs 32.8% (HR 0.78, 0.66–0.92; P = 0.003), consistent across disease-risk and conditioning-intensity subgroups.<br>No significant difference in NRM, OS, PFS, GRFS, or the incidence and severity of acute or chronic GvHD.<br>Authors conclude PTCy can be recommended in matched related donor transplantation.
**Penack 2024** [14]	EBMT registry, retrospective	Mismatched unrelated donor	PTCy vs ATG	severe acute GVHD grades III–IV : PTCy vs ATG: 11.4% vs. 13.8%; HR: 0.78 [95% CI 0.59–1.05], *p* = 0.1<br><br>2-year cGVHD PTCy vs ATG 31.7% vs. 30.3%; HR 0.95 [95% CI 0.74–1.22], p = 0.67)	2-year NRM 18% PTCy vs. 24.9% ATG; HR: 0.74 [95% CI 0.56–0.97], *p* = 0.028<br><br>2-year Relapse PTCy vs. rATG arm 22.9% vs. 26.2%; HR: 0.82 [95% CI 0.67–1.01], p = 0.068	2-year OS PTCy vs ATG: 65.7% vs. 55.7%; HR: 0.77 [95% CI 0.65–0.90], *p* < 0.001)<br><br>2year PFS (PTCy vs ATG) 59.1% vs. 48.8%; HR: 0.78 [95% CI 0.67–0.91], *p* = 0.001	
**Brissot 2024**[15]	Phase II RCT, open-label<br>n = 89	Matched sibling or 10/10 matched unrelated; RIC (Flu-Bu2); PBSC	PTCy vs ATG	—	—	1-year OS PTCy 79.5% (95% CI, 64.4–88.8) vs. ATG 73.3% (95% CI, 57.8–83.9), (P = 0.41).<br><br>1-year DFS PTCy 65.9% (95% CI, 50–77.8) vs.ATG 62.2% (95% CI, 46.5–74.6) (*P* = 0.5)	1-year GRFS 54.5% vs 43.2% (.27, NS); 1-year DFS 65.9% vs 67.6% (P = .99); relapse, NRM, OS comparable. Neutrophil and platelet recovery significantly slower with PTCy. Underpowered.
**Robin 2018 [16]**	Retrospective ATG vs Alemtuzumab matched to HLA, age, gender<br><br>N= 238 (119 per group)	Adult patients with myeloid disease<br><br>119 each group	ATG vs Campath	Grade II-IV aGVHD at 6mth 44% ATG vs 15% campath 9p<0.0001)<br><br>Extensive cGVHD at 2-year 26% ATg vs 14% campath (P = 0.003)	5-year CI relapse 45% campath vs 35% ATG (P = 0.022)<br><br>5-year TRM 19% campath 21% ATG (P = 0.48)	5-year OS 47% (95% CI 36.5-60.5) campth vs 48% (95% CI 40.7-56.6) (P = 0.92 log rank)	Retrospective study but demonstrated lower rates of GvHD with campath compared to ATG however higher relapse
**Bug 2026**<br><br>**[17]**	ATG vs Alemtuzumab in ALL<br><br>n=357	Adults with ALL in CR1	Campath n=90 vs ATG n=90	CI severe aGVHD 8% alemtuzuma vs 6.8% ATG (HR 1.11, P = 0.84)<br><br>Extensie cGVHD Alemtuzumabt 8.4% vs 12.5% (HR 0.5, P = 0.15)	NRM alemtuzumab 19.9% vs 25.4% HR 0.75, P = 0.32	2-year LFS 56.4% Alemtuzumab vs ATG 50.6 HR 0.82, P = 0.34<br><br>OS 62.7% alemtuzumab vs 62.9% HR 0.91, P = 0.67	

Trial (ref)	Design, n	Population / donor	Intervention	GvHD	Relapse & NRM	Survival	Key limitations / interpretive considerations
**B. Ex vivo graft engineering**							
**B1. CD34+ selection and pan-T-cell depletion**							
**Aversa 2005** [18]	Phase II single-arm<br>n = 104<br><br>(67 AML, 37 ALL)	Adults, high-risk acute leukaemia; haploidentical; TBI/thiotepa/fludarabine/ATG	Megadose CD34+ immunoselection; no post-transplant GvHD prophylaxis	aGvHD 8/100<br>cGvHD 5/70 evaluable	Relapse 9/66 transplanted in remission; 17/38 in relapse<br>38 deaths from non-leukaemic causes	EFS 48% ± 8% (AML in remission, n = 42); 46% ± 10% (ALL in remission, n = 24)	Low GvHD rates, but substantial non-relapse mortality associated with profound immune depletion.
**Pasquini 2012 — BMT CTN 0303 comparative analysis** [19]	Non-randomised contemporaneous comparative study; 44 TCD vs 84 IST	Adults, AML CR1/CR2; HLA-identical sibling; myeloablative	CD34 selection (CliniMACS) as sole immunosuppression	100-d grade II–IV aGvHD 23% vs 39%, P = 0.07<br>2-year cGvHD 19% vs 50%, *P* < .001	No difference in relapse or TRM	2-year GvHD-free survival 41% vs 19%, P = 0.006<br>No difference in DFS or OS	Not randomised. On immunosuppression at 1 year: 12% vs 54%. Morbidity benefit without survival benefit.
**B2. TCRαβ/CD19 and CD3/CD19 depletion**							
**αβ/CD19 depletion, paediatric**<br>Merli 2024 [20]	Phase I–II, updated final analysis<br>n = 213 children (from original 80)	Children, acute leukaemia; haploidentical	TCRαβ/CD19 depletion	Grade II–IV aGvHD 14.7%<br>cGvHD 8.1%	5-year relapse 22.7% (16.9–29.2)<br>5-year NRM 5.2% (2.8–8.8)	10-year OS 75.4% (68.6–80.9)<br>10-year DFS 71.6% (64.4–77.6)	Largest and longest-followed dataset for the platform (median FU 47.6 mo). Outcomes near-superimposable on matched-donor HSCT. Paediatric only.
**αβ/CD19 depletion, adults + children**<br>Bethge 2022[21]	Phase I/II multicentre<br>n = 60 (30 adult, 30 paediatric)	Mixed malignant (n = 5) and non-malignant (n = 4) + solid tumours (n = 6); haploidentical; RIC (flu/thiotepa/melphalan/ATG)	TCRαβ/CD19 depletion, decentralised manufacture at 6 sites	No grade III/IV aGvHD<br>Grade II aGvHD 10% (6 patients)	NRM: 5% d100, 15% 1-year, 17% 2-year	2-year OS 63%<br>2-year DFS 50%	Key adult evidence for this platform. Small, heterogeneous, includes non-malignant cases .
**CD3/CD19 depletion**<br>Federmann 2012 [22]	Phase II multicentre<br>n = 61	Adults (median 46 y), high risk haploidentical; RIC (flu/thiotepa/melphalan)	CD3/CD19 depletion + OKT-3	Grade II–IV aGvHD 46%<br>cGvHD 18%	2-year relapse/progression 31%<br>NRM 23% d100, 41% 2-year	1-year OS 41%<br>2-year OS 28%	Worst outcomes in the table — but an exceptionally high-risk population (half not in remission). Illustrates that platform choice cannot be separated from patient selection.
**B3. CD45RA and naïve T-cell depletion**							
**Naïve T-cell depletion** — Bleakley 2015 [23]]	Phase I/II, n=35	Adults with high-risk acute leukaemia; HLA-matched related/unrelated donors	CD34 selection + CD45RA-depleted memory T-cell add-back	Grade II–IV aGVHD 66%; cGVHD 9%	2-year relapse 21% (95% CI 7–35)<br>2-year NRM 9% (95% CI 0–19)	2-year OS 78%	single-arm first-in-human study; high incidence of predominantly steroid-responsive gastrointestinal grade II aGVHD

Trial (ref)	Design, n	Population / donor	Intervention	GvHD	Relapse & NRM	Survival	Key limitations / interpretive considerations
**Naïve T-cell depletion**<br>Bleakley 2022[24]	Three pooled phase II trials<br>n = 138	Adults + children, acute leukaemia; HLA-matched related or unrelated; high/intermediate-dose TBI	CD34 selection + defined dose of CD45RA-depleted memory T cells	3-year cGvHD 7% (2–11); moderate 1%, severe 0%<br>Grade III aGvHD 4% (1–8); grade IV 0%<br>Grade II aGvHD 71% (mostly stage 1 upper GI)	3-year relapse 23% (16–30)<br>3-year NRM 8% (3–13)	3-year OS 77% (71–85)<br>3-year cGvHD-free relapse-free survival 68% (61–76)	Single-arm; matched donors only. Very low chronic and severe acute GVHD, but grade II acute GVHD remained frequent and was mostly mild upper-GI disease; no randomised comparator.
**CD45RA depletion + NK add-back**<br>Naik 2024[25]	Phase II<br>n = 72	Paediatric/young patients, high-risk haematological malignancy; haploidentical	CD34-enriched/CD45RA-depleted graft + NK cell add-back; short-course sirolimus or MMF; serotherapy-free submyeloablative conditioning	1-year aGvHD II–IV 33.3%; grade III–IV 29.2%<br>3-year cGVHD 25.6%; moderate–severe 15.6%	3-year relapse 26.8%<br>3-year NRM 11.5%	3-year OS 72.5; EFS 62.2% In morphological CR (n=53): OS 86.4, EFS 77.4%	Multi-component platform (CD34 enrichment, CD45RA depletion, NK-cell add-back, conditioning and short-course prophylaxis) with heterogeneous disease status; outcomes cannot be attributed to CD45RA depletion alone. Included patients with active disease and detectable MRD, making the relapse rate more impressive than it appears.
**B4. Regulatory and conventional T-cell (precision-engineered) grafts**							
**Treg/Tcon adoptive immunotherapy**<br>Martelli 2014 [26]	Phase II<br>n = 43	Adults, high-risk acute leukaemia (33 AML, 10 ALL); haploidentical; TBI-based	CD34 megadose + Tregs then Tcons; **no** post-transplant immunosuppression	≥grade 2 aGvHD 15%	Cumulative incidence of relapse 0.05 (5%) — significantly better than historical controls	DFS 0.56 at median FU 46 months	The lowest relapse rate in the table. The key evidence that GvL can be preserved while GvHD is suppressed.
**Precision-T**<br>[27]	Phase III RCT<br>n = 187	Adults, acute leukaemia or MDS; HLA-matched; myeloablative; G-CSF PBSC	2 arms: Orca-T + tacrolimus vs conventional allograft + Tac/MTX	Mod–severe cGvHD 12.6% vs 44.0% (Gray *P* < .001)	NRM 3.4% vs 13.2% (P = 0.03)<br>1-year cumulative incidence of relapse/progression 21.1% vs 12.7%	1-year cGFS (primary) 78.0% vs 38.4%, HR 0.26 (0.14–0.47), *P* < .001<br>1-year GRFS 63.1% vs 30.9%, *P* < .001<br>1-year OS 93.9% vs 83.1%, P = .12 (NS)	First positive phase III for graft engineering. OS difference not significant Comparator was Tac/MTX, *not* PTCy.
**B5. Adoptive add-back and suicide-gene strategies**							
**αβ-TCD + memory T add-back, adults**<br>Koh 2026 [28]	Prospective multicentre single-arm + propensity-matched comparator<br>n = 145; propensity-matched analysis 53 vs 53	Adults, mixed haematological malignancy; haploidentical	αβ T-cell depletion + upfront CD45RA-depleted memory T-cell DLI	180-d grade II–IV aGvHD 31%; grade III–IV 8%<br>2-year cGvHD 4%	2-year relapse 22%<br>2-year NRM 17**%**	estimated 3-year OS 67%<br>estimated 3-year EFS 62%<br>estimated 3-year GRFS 59%	Prospective single-arm platform study with a propensity-matched PTCy comparator; Not randomised.

Trial (ref)	Design, n	Population / donor	Intervention	GvHD	Relapse & NRM	Survival	Key limitations / interpretive considerations
**Memory DLI after αβ-TCD** Dunaikina/Maschan 2021 [29]	RCT n = 149 children (76 vs 73)	Children, high-risk leukaemia; haploidentical; TBI or treosulfan; ATG replaced by tocilizumab + abatacept	Scheduled low-dose CD45RA-depleted DLI vs standard care, both after αβ T-cell-depleted haplo	Grade II–IV aGvHD 14% vs 12%, P = 0.8 (NS)	Whole cohort 2-year: relapse 25%, NRM 2% No difference between arms	Whole cohort 2-year: EFS 71%, OS 80% No difference between arms	Neither primary endpoint differed significantly (aGvHD, CMV viraemia 45% vs 55%, *P* = .4). Benefit limited to improved CMV-specific T-cell recovery. Excellent whole-cohort outcomes.
**CD8-depleted DLI** Soiffer 2002 [30]	RCT n = 18 (9 vs 9)	Adults in remission, prophylactic DLI at 6 months	Unselected DLI vs CD8-depleted DLI, both after TCD allo-BMT	aGvHD 6/9 (67%) unselected vs 0/9 (0%) depleted, P = 0.009	Relapse 3 vs 1 Toxic deaths 2 vs 0	2-year RFS 88% vs 40%, P = .08	Very small (n = 18). Historically important as an early randomised test of cell-product engineering.
**Suicide-gene DLI (TK007)** Ciceri 2009[31]	Phase I–II multicentre n = 50 transplanted, 28 received TK-cells	Adults (median 51 y), high-risk leukaemia; haploidentical	Donor lymphocytes transduced with HSV thymidine kinase; ganciclovir as the "off switch"	aGvHD grade I–IV in 10; cGvHD in 1 — all controlled by suicide-gene induction	Only 1 infectious death beyond day 100 (at 166 d)	3-year OS 49% (25–73) in the 19 patients in remission at transplant	Primary endpoint was immune reconstitution: 22/28 achieved it at median 75 d post-transplant.
**Photodepleted DLI (ATIR101)** Roy 2020[32]	Phase II + observational comparators n = 23 ATIR101; comparators: TCD-haplo alone 35, MUD 64, MMUD 37, UCB 22	Adults, acute leukaemia; haploidentical; **no** post-transplant immunosuppression	CD34-selected graft + donor lymphocytes photodepleted of recipient-alloreactive T cells	Not stated in abstract	6-mo NRM 13% (primary endpoint) 1-year NRM lower than TCD-haplo alone, P = 0.008	1-year GRFS 56.5% vs MMUD 27.0% and UCB 22.7% (both *P* < .03); vs MUD 40.6% (NS)	Comparators are observational, not randomised. Confirmatory phase III (HATCY) vs PTCy was terminated; product not approved.
**B6. Gene-edited grafts**							
**Gene-edited graft (trem-cel)** [33]	Phase 1/2a open-label, 15 sites n = 30 (19 received GO maintenance)	Adults, high-risk AML/MDS; myeloablative	CRISPR–Cas9 CD33-deleted donor CD34+ cells, then gemtuzumab ozogamicin maintenance (0.5–2.0 mg/m²)	Reported as secondary endpoint; not quantified in abstract	3 transplant-related deaths (renal failure, sepsis, sinusoidal obstruction syndrome)	Not reported	100% (30/30) met primary endpoint of neutrophil engraftment by day 28, median 10 days (95% CI 9–10). GO tolerated to 2 mg/m² with no prolonged high-grade cytopenias. Trial stopped early; this is the final report.
**C. Direct comparisons of ex vivo engineering against in vivo T-cell depletion**							
**BMT CTN 1301 (PROGRESS II)** Luznik 2022[34]	Phase III RCT, 3 arms 346 enrolled, 327 transplanted, 300 per-protocol	Adults, acute leukaemia or MDS; HLA-matched; myeloablative	3 arms: (1) CD34-selected PBSC (2) PTCy + BM (3) Tac/MTX + BM (control)	Mod–severe cGvHD, CD34 vs control: HR 0.25 (0.12–0.52), P = 0.02	PTCy relapse HR 0.52 (0.28–0.96), P = .037 TRM, CD34 vs control: HR 2.76 (1.26–6.06), P = 0.01	2-year CRFS: 50.6% CD34 (HR 0.80, 0.56–1.15, P = .24); 48.1% PTCy (HR 0.86, P = .41); 41.0% control 2-year OS: 60.1% CD34 (HR 1.74, 1.09–2.80, P = .02); 76.2% PTCy; 76.1% control	Negative trial. Neither CNI-free arm beat standard care. CD34 selection reduced cGvHD but had *worse* OS and higher TRM.

Trial (ref)	Design, n	Population / donor	Intervention	GvHD	Relapse & NRM	Survival	Key limitations / interpretive considerations
**Lum 2024**<br>EBMT Inborn Errors Working Party<br>[35]	Retrospective multicentre registry<br>n = 306 (TCRαβ 167, PTCy 139)<br>first HSCT 2010–2019	Children, inborn errors of immunity (non-malignant)<br>median age 1.2-year (0.03–19.6)<br>HLA-mismatched donor	TCRαβ/CD19 depletion vs PTCy<br>ex vivo versus in vivo, head-to-head	Severe aGvHD 6% (2–9) TCRαβ vs 15% (9–21) PTCy, P = 0.007<br>cGvHD 7% (3–11) vs 11% (6–17), P = 0.173 (NS)	No relapse endpoint (non-malignant).<br>Toxicity splits by platform. PTCy: VOD 14.4% vs 4.9% (P = 0.009), AKI 12.7% vs 4.6% (P = 0.032), pulmonary 38.2% vs 24.1% (P = *0*.017). TCRαβ: adenoviraemia 18.3% vs 8.0% (P = 0.015), primary graft failure 10% vs 5% (P = 0.048), second HSCT 17.4% vs 7.9% (P = 0.023)	3-year OS 78% (71–84) vs 66% (57–74), P = .013<br>3-year EFS 58% (50–66) vs 57% (48–66), *P* = .804<br>3-year GvHD-free EFS 53% (44–61) vs 41% (32–50), *P* = .080	The OS difference is univariate only. On multivariable analysis only pre-HSCT morbidity score (HR 2.27, 1.07–4.80, *P* = .032) and non-busulfan/treosulfan conditioning (HR 3.12, 1.98–4.92, *P* < .001) independently predicted survival; platform did not, and EFS was identical. Authors conclude both are suitable with different advantages.
**Nalla 2026**<br>Apollo Hospitals, Chennai[36]	Retrospective single-centre<br>n = 105 (TCRαβ 65, PTCy 40)<br>transplants 2009–2024, min 12 mo follow-up	Children, inborn errors of immunity (non-malignant)<br>birth–18 year<br>haploidentical donor	TCRαβ/CD19 depletion vs T-replete graft + PTCy<br>ex vivo versus in vivo, head to head	No significant difference.<br>aGvHD 8.6% TCRαβ vs 15% PTCy; cGvHD 12% vs 17.5%<br>Engraftment 89% vs 85%	No relapse endpoint (non-malignant).<br>Viral reactivation reported as the persisting challenge; authors propose letermovir prophylaxis and memory cell add-back	5-year OS 62% (48–73) TCRαβ vs 54% PTCy<br>Age <2-year: 60% vs 37%, P = 0.001	Weakest evidence in this section. Single centre, retrospective, spanning 15 years so subject to era effects. No P value is reported for the overall OS comparison, only for the under-2 subgroup, which is a subgroup analysis. The published abstract gives an identical 95% CI (48–73) for both OS estimates, which is an evident transcription error; Non-malignant paediatric cohort, comparator only.
**D. Systematic reviews and meta-analyses**							
**Arcuri 2026**<br>[37]	Meta-analysis of 16 randomised trials<br>1,344 intervention vs 1,260 control	Sibling or unrelated donors	ATG/ATLG, PTCy, sirolimus, abatacept or alemtuzumab added to standard prophylaxis	—		—	Meta-analysis of intensification strategies added to standard prophylaxis, rather than a direct comparison between individual agents. No significant effect on relapse (HR 1.12, P = 0.16) or PFS (HR 0.92, P = 0.16). Sensitivity analysis flagged one alemtuzumab outlier with higher relapse, and a modest increased relapse risk with ATLG. Concludes that adding ATG, sirolimus, abatacept or PTCy does not increase relapse.
**Bentolila 2026**<br>[38]	Network meta-analysis of randomised trials	Matched sibling donor	Comparison of GvHD prophylaxis regimens	—	—	—	Directly on point for optimal prophylaxis in sibling transplant.

Trial (ref)	Design, n	Population / donor	Intervention	GvHD	Relapse & NRM	Survival	Key limitations / interpretive considerations
**Jin 2025** [39]	Systematic review and meta-analysis, 9 studies, n = 1,674	Haploidentical	PTCy vs ATG	Grade II-IV aGVHD PTCy vs ATG HR 0.81 (95% CI 0.62–1.05), with *P* = 0.108 (Fig. 3), severe cGVHD PTCy vs ATG (RR = 1.22, 95% CI 0.51–2.88, P = 0.658; I^2 = 0%, P = 0.414)	NRM PTCy vs ATG HR = 0.67, 95% CI 0.4–1.11, *P* = 0.117;I^2 = 72% and *P* = 0.003) Relapse PTCy vs ATG (HR = 0.69, 95% CI 0.53–0.9, *P* = 0.007; I^2 = 0.0%, *P* = 0.801)	OS PTCy vs ATG HR = 0.7, 95% CI 0.51–0.97, *P* = 0.032; I^2 = 59.7%, *P* = 0.011) LFS PTCy vs ATG (HR = 0.66, 95% CI 0.53–0.81, *P* = 0.000) (Fig. 6). With I^2 = 0.0% and *P* = 0.563	Similar efficacy for PTCy and ATG in GVHD prevention however retrospective evidence and heterogeneity limit meaningful comparison. PTCy superior to ATG for OS and relapse.

Abbreviations: aGVHD, acute graft-versus-host disease; AKI, acute kidney injury; ALL, acute lymphoblastic leukaemia; AML, acute myeloid leukaemia; ATG, anti-thymocyte globulin; ATG-F, anti-T-cell globulin Fresenius; ATLG, anti-T-lymphocyte globulin; BM, bone marrow; BMT CTN, Blood and Marrow Transplant Clinical Trials Network; cGFS, chronic GVHD-free survival; cGVHD, chronic graft-versus-host disease; CI, confidence interval; CNI, calcineurin inhibitor; CR, complete remission; CR1/CR2, first/second complete remission; CRFS, chronic GVHD/relapse-free survival; CRISPR–Cas9, clustered regularly interspaced short palindromic repeats/CRISPR-associated protein 9; CsA, cyclosporin A; DFS, disease-free survival; DLI, donor lymphocyte infusion; EBMT, European Society for Blood and Marrow Transplantation; EFS, event-free survival; G-CSF, granulocyte colony-stimulating factor; GO, gemtuzumab ozogamicin; GRFS, GVHD-free, relapse-free survival; GVHD, graft-versus-host disease; GVL, graft-versus-leukaemia; HLA, human leukocyte antigen; HR, hazard ratio; HSCT, haematopoietic stem cell transplantation; HSV, herpes simplex virus; IST, immunosuppressive therapy; LFS, leukaemia-free survival; MAC, myeloablative conditioning; MDS, myelodysplastic syndrome; MMF, mycophenolate mofetil; MMUD, mismatched unrelated donor; MPA, mycophenolic acid; MPN, myeloproliferative neoplasm; MRD, measurable residual disease; MTX, methotrexate; MUD, matched unrelated donor; NK, natural killer; NMA, non-myeloablative conditioning; NRM, non-relapse mortality; OR, odds ratio; OS, overall survival; PBSC, peripheral blood stem cells; PFS, progression-free survival; PTCy, post-transplant cyclophosphamide; RCT, randomised controlled trial; RFS, relapse-free survival; RIC, reduced-intensity conditioning; RR, risk ratio; TBI, total body irradiation; TCD, T-cell depletion; Tcon, conventional T cell; TCRαβ, αβ T-cell receptor; Treg, regulatory T cell; TRM, transplant-related mortality; UCB, umbilical cord blood; VOD, veno-occlusive disease.

References

1. Finke, J.; Bethge, W.A.; Schmoor, C.; Ottinger, H.D.; Stelljes, M.; Zander, A.R.; Volin, L.; Ruutu, T.; Heim, D.A.; Schwerdtfeger, R.; et al. Standard graft-versus-host disease prophylaxis with or without anti-T-cell globulin in haematopoietic cell transplantation from matched unrelated donors: a randomised, open-label, multicentre phase 3 trial. *Lancet Oncol* **2009**, *10*, 855–864, doi:10.1016/s1470-2045(09)70225-6.
2. Soiffer, R.J.; Kim, H.T.; McGuirk, J.; Horwitz, M.E.; Johnston, L.; Patnaik, M.M.; Rybka, W.; Artz, A.; Porter, D.L.; Shea, T.C.; et al. Prospective, Randomized, Double-Blind, Phase III Clinical Trial of Anti-T-Lymphocyte Globulin to Assess Impact on Chronic Graft-Versus-Host Disease-Free Survival in Patients Undergoing HLA-Matched Unrelated Myeloablative Hematopoietic Cell Transplantation. *J Clin Oncol* **2017**, *35*, 4003–4011, doi:10.1200/jco.2017.75.8177.
3. Kröger, N.; Solano, C.; Wolschke, C.; Bandini, G.; Patriarca, F.; Pini, M.; Nagler, A.; Selleri, C.; Risitano, A.; Messina, G.; et al. Antilymphocyte Globulin for Prevention of Chronic Graft-versus-Host Disease. *N Engl J Med* **2016**, *374*, 43–53, doi:10.1056/NEJMoa1506002.
4. Walker, I.; Panzarella, T.; Couban, S.; Couture, F.; Devins, G.; Elemary, M.; Gallagher, G.; Kerr, H.; Kuruvilla, J.; Lee, S.J.; et al. Addition of anti-thymocyte globulin to standard graft-versus-host disease prophylaxis versus standard treatment alone in patients with haematological malignancies undergoing transplantation from unrelated donors: final analysis of a randomised, open-label, multicentre, phase 3 trial. *Lancet Haematol* **2020**, *7*, e100–e111, doi:10.1016/s2352-3026(19)30220-0.
5. Kottaridis, P.D.; Milligan, D.W.; Chopra, R.; Chakraverty, R.K.; Chakrabarti, S.; Robinson, S.; Peggs, K.; Verfuerth, S.; Pettengell, R.; Marsh, J.C.; et al. In vivo CAMPATH-1H prevents graft-versus-host disease following nonmyeloablative stem cell transplantation. *Blood* **2000**, *96*, 2419–2425.
6. Chakraverty, R.; Peggs, K.; Chopra, R.; Milligan, D.W.; Kottaridis, P.D.; Verfuerth, S.; Geary, J.; Thuraisundaram, D.; Branson, K.; Chakrabarti, S.; et al. Limiting transplantation-related mortality following unrelated donor stem cell transplantation by using a nonmyeloablative conditioning regimen. *Blood* **2002**, *99*, 1071–1078, doi:10.1182/blood.v99.3.1071.
7. Luznik, L.; O'Donnell, P.V.; Symons, H.J.; Chen, A.R.; Leffell, M.S.; Zahurak, M.; Gooley, T.A.; Piantadosi, S.; Kaup, M.; Ambinder, R.F.; et al. HLA-haploidentical bone marrow transplantation for hematologic malignancies using nonmyeloablative conditioning and high-dose, posttransplantation cyclophosphamide. *Biol Blood Marrow Transplant* **2008**, *14*, 641–650, doi:10.1016/j.bbmt.2008.03.005.
8. Bolaños-Meade, J.; Hamadani, M.; Wu, J.; Al Malki, M.M.; Martens, M.J.; Runaas, L.; Elmariah, H.; Rezvani, A.R.; Gooptu, M.; Larkin, K.T.; et al. Post-

Transplantation Cyclophosphamide-Based Graft-versus-Host Disease Prophylaxis. *N Engl J Med* **2023**, *388*, 2338–2348, doi:10.1056/NEJMoa2215943.
9. Curtis, D.J.; Patil, S.S.; Reynolds, J.; Purtill, D.; Lewis, C.; Ritchie, D.S.; Gottlieb, D.J.; Yeung, D.T.; Wong, E.; Tey, S.K.; et al. Graft-versus-Host Disease Prophylaxis with Cyclophosphamide and Cyclosporin. *N Engl J Med* **2025**, *393*, 243–254, doi:10.1056/NEJMoa2503189.
10. Broers, A.E.C.; de Jong, C.N.; Bakunina, K.; Hazenberg, M.D.; van Marwijk Kooy, M.; de Groot, M.R.; van Gelder, M.; Kuball, J.; van der Holt, B.; Meijer, E.; et al. Posttransplant cyclophosphamide for prevention of graft-versus-host disease: results of the prospective randomized HOVON-96 trial. *Blood Adv* **2022**, *6*, 3378–3385, doi:10.1182/bloodadvances.2021005847.
11. Al Malki, M.M.; Bo-Subait, S.; Logan, B.; Olson, J.; Kou, J.; Smith, S.; Leckrone, E.; Wu, J.; Stefanski, H.E.; Auletta, J.J.; et al. Post-Transplant Cyclophosphamide-Based Graft-Versus-Host Disease Prophylaxis After Mismatched Unrelated Donor Peripheral Blood Stem Cell Transplantation. *J Clin Oncol* **2025**, *43*, 2772–2781, doi:10.1200/jco-25-00856.
12. Schetelig, J.; Stelljes, M.; Kunadt, D.; Schröder, T.; Müller, L.P.; Stölzel, F.; Bug, G.; Jost, E.; Morgner, A.; Lakner, J.; et al. GVHD prophylaxis including ATG remains standard of care for HLA-compatible unrelated donor hematopoietic cell transplantation: results from a large randomized controlled trial comparing ATG and PTCy. In Proceedings of the European Hematology Association (EHA) Congress 2026, Stockholm, Sweden, June 2026, 2026.
13. Penack, O.; Abouqateb, M.; Peczynski, C.; Boreland, W.; Benakli, M.; Kröger, N.; Blau, I.W.; Blaise, D.; Sanz, J.; Eder, M.; et al. GVHD prophylaxis in matched related stem cell transplantation: Why post-transplant cyclophosphamide can be recommended a study by the EBMT transplant complications working party. *Leukemia* **2025**, *39*, 1503–1511, doi:10.1038/s41375-025-02619-1.
14. Penack, O.; Abouqateb, M.; Peczynski, C.; Boreland, W.; Gülbas, Z.; Gedde-Dahl, T.; Castilla-Llorente, C.; Kröger, N.; Eder, M.; Rambaldi, A.; et al. PTCy versus ATG as graft-versus-host disease prophylaxis in mismatched unrelated stem cell transplantation. *Blood Cancer J* **2024**, *14*, 45, doi:10.1038/s41408-024-01032-8.
15. Brissot, E.; Labopin, M.; Labussière, H.; Fossard, G.; Chevallier, P.; Guillaume, T.; Yakoub-Agha, I.; Srour, M.; Bulabois, C.E.; Huynh, A.; et al. Post-transplant cyclophosphamide versus anti-thymocyte globulin after reduced intensity peripheral blood allogeneic cell transplantation in recipients of matched sibling or 10/10 HLA matched unrelated donors: final analysis of a randomized, open-label, multicenter, phase 2 trial. *Blood Cancer J* **2024**, *14*, 31, doi:10.1038/s41408-024-00990-3.
16. Robin, M.; Raj, K.; Chevret, S.; Gauthier, J.; de Lavallade, H.; Michonneau, D.; McLornan, D.; Peffault de Latour, R.; Potter, V.; Kulasekararaj, A.; et al. Alemtuzumab vs anti-thymocyte globulin in patients transplanted from an unrelated donor after a reduced intensity conditioning. *Eur J Haematol* **2018**, *101*, 466–474, doi:10.1111/ejh.13085.
17. Bug, G.; Labopin, M.; Byrne, J.L.; Mielke, S.; Orchard, K.; Paneesha, S.; Potter, V.; Blaise, D.; Besley, C.; Snowden, J.A.; et al. Anti-thymocyte globulin (ATG)- or alemtuzumab-based graft-versus-host disease prophylaxis in reduced-intensity conditioning allogeneic hematopoietic cell transplantation (HCT) for patients 40

years and older with acute lymphoblastic leukemia in first complete remission: a study from the EBMT Acute Leukemia Working Party. *Bone Marrow Transplantation* **2026**, *61*, 462–468, doi:10.1038/s41409-026-02805-4.
18. Aversa, F.; Terenzi, A.; Tabilio, A.; Falzetti, F.; Carotti, A.; Ballanti, S.; Felicini, R.; Falcinelli, F.; Velardi, A.; Ruggeri, L.; et al. Full haplotype-mismatched hematopoietic stem-cell transplantation: a phase II study in patients with acute leukemia at high risk of relapse. *J Clin Oncol* **2005**, *23*, 3447–3454, doi:10.1200/jco.2005.09.117.
19. Pasquini, M.C.; Devine, S.; Mendizabal, A.; Baden, L.R.; Wingard, J.R.; Lazarus, H.M.; Appelbaum, F.R.; Keever-Taylor, C.A.; Horowitz, M.M.; Carter, S.; et al. Comparative outcomes of donor graft CD34+ selection and immune suppressive therapy as graft-versus-host disease prophylaxis for patients with acute myeloid leukemia in complete remission undergoing HLA-matched sibling allogeneic hematopoietic cell transplantation. *J Clin Oncol* **2012**, *30*, 3194–3201, doi:10.1200/jco.2012.41.7071.
20. Merli, P.; Algeri, M.; Galaverna, F.; Bertaina, V.; Lucarelli, B.; Boccieri, E.; Becilli, M.; Quagliarella, F.; Rosignoli, C.; Biagini, S.; et al. TCRαβ/CD19 cell-depleted HLA-haploidentical transplantation to treat pediatric acute leukemia: updated final analysis. *Blood* **2024**, *143*, 279–289, doi:10.1182/blood.2023021336.
21. Bethge, W.A.; Eyrich, M.; Mielke, S.; Meisel, R.; Niederwieser, D.; Schlegel, P.G.; Schulz, A.; Greil, J.; Bunjes, D.; Brecht, A.; et al. Results of a multicenter phase I/II trial of TCRαβ and CD19-depleted haploidentical hematopoietic stem cell transplantation for adult and pediatric patients. *Bone Marrow Transplant* **2022**, *57*, 423–430, doi:10.1038/s41409-021-01551-z.
22. Federmann, B.; Bornhauser, M.; Meisner, C.; Kordelas, L.; Beelen, D.W.; Stuhler, G.; Stelljes, M.; Schwerdtfeger, R.; Christopeit, M.; Behre, G.; et al. Haploidentical allogeneic hematopoietic cell transplantation in adults using CD3/CD19 depletion and reduced intensity conditioning: a phase II study. *Haematologica* **2012**, *97*, 1523–1531, doi:10.3324/haematol.2011.059378.
23. Bleakley, M.; Heimfeld, S.; Loeb, K.R.; Jones, L.A.; Chaney, C.; Seropian, S.; Gooley, T.A.; Sommermeyer, F.; Riddell, S.R.; Shlomchik, W.D. Outcomes of acute leukemia patients transplanted with naive T cell-depleted stem cell grafts. *J Clin Invest* **2015**, *125*, 2677–2689, doi:10.1172/jci81229.
24. Bleakley, M.; Sehgal, A.; Seropian, S.; Biernacki, M.A.; Krakow, E.F.; Dahlberg, A.; Persinger, H.; Hilzinger, B.; Martin, P.J.; Carpenter, P.A.; et al. Naive T-Cell Depletion to Prevent Chronic Graft-Versus-Host Disease. *J Clin Oncol* **2022**, *40*, 1174–1185, doi:10.1200/jco.21.01755.
25. Naik, S.; Li, Y.; Talleur, A.C.; Selukar, S.; Ashcraft, E.; Cheng, C.; Madden, R.M.; Mamcarz, E.; Qudeimat, A.; Sharma, A.; et al. Memory T-cell enriched haploidentical transplantation with NK cell addback results in promising long-term outcomes: a phase II trial. *J Hematol Oncol* **2024**, *17*, 50, doi:10.1186/s13045-024-01567-0.
26. Martelli, M.F.; Di Ianni, M.; Ruggeri, L.; Falzetti, F.; Carotti, A.; Terenzi, A.; Pierini, A.; Massei, M.S.; Amico, L.; Urbani, E.; et al. HLA-haploidentical transplantation with regulatory and conventional T-cell adoptive immunotherapy prevents acute leukemia relapse. *Blood* **2014**, *124*, 638–644, doi:10.1182/blood-2014-03-564401.

27. Meyer, E.H.; Salhotra, A.; Gandhi, A.P.; Pantin, J.; Patel, S.S.; Hoeg, R.T.; Gomez-Arteaga, A.; Faramand, R.; Tamari, R.; Waller, E.K.; et al. Orca-T vs allogeneic hematopoietic stem cell transplantation (Precision-T): a multicenter, randomized phase 3 trial. *Blood* **2026**, *147*, 1168–1177, doi:10.1182/blood.2025031313.
28. Koh, L.P.; Linn, Y.C.; Boo, Y.L.; Ling, V.; Lim, Z.Y.; Poon, M.; Quek, J.; Than, H.; Diong, C.P.; Vellayappan, B.; et al. Upfront memory T cell add-back with haploidentical TCRαβ-depleted graft in adults with haematological malignancies: a nationwide, multicentre, single-arm, prospective study. *Bone Marrow Transplant* **2026**, *61*, 26–35, doi:10.1038/s41409-025-02728-6.
29. Dunaikina, M.; Zhekhovtsova, Z.; Shelikhova, L.; Glushkova, S.; Nikolaev, R.; Blagov, S.; Khismatullina, R.; Balashov, D.; Kurnikova, E.; Pershin, D.; et al. Safety and efficacy of the low-dose memory (CD45RA-depleted) donor lymphocyte infusion in recipients of αβ T cell-depleted haploidentical grafts: results of a prospective randomized trial in high-risk childhood leukemia. *Bone Marrow Transplant* **2021**, *56*, 1614–1624, doi:10.1038/s41409-021-01232-x.
30. Soiffer, R.J.; Alyea, E.P.; Hochberg, E.; Wu, C.; Canning, C.; Parikh, B.; Zahrieh, D.; Webb, I.; Antin, J.; Ritz, J. Randomized trial of CD8+ T-cell depletion in the prevention of graft-versus-host disease associated with donor lymphocyte infusion. *Biol Blood Marrow Transplant* **2002**, *8*, 625–632, doi:10.1053/bbmt.2002.v8.abbmt080625.
31. Ciceri, F.; Bonini, C.; Stanghellini, M.T.; Bondanza, A.; Traversari, C.; Salomoni, M.; Turchetto, L.; Colombi, S.; Bernardi, M.; Peccatori, J.; et al. Infusion of suicide-gene-engineered donor lymphocytes after family haploidentical haemopoietic stem-cell transplantation for leukaemia (the TK007 trial): a non-randomised phase I-II study. *Lancet Oncol* **2009**, *10*, 489–500, doi:10.1016/s1470-2045(09)70074-9.
32. Roy, D.C.; Walker, I.; Maertens, J.; Lewalle, P.; Olavarria, E.; Selleslag, D.; Lachance, S.; Buyse, M.; Wang, K.; Rovers, J.; et al. ATIR101 administered after T-cell-depleted haploidentical HSCT reduces NRM and improves overall survival in acute leukemia. *Leukemia* **2020**, *34*, 1907–1923, doi:10.1038/s41375-020-0733-0.
33. DiPersio, J.F.; Koehne, G.; Shah, N.N.; Bernard, L.; Suh, H.C.; Koura, D.; Tamari, R.; Mushtaq, M.U.; Maakaron, J.; Rimando, J.; et al. CRISPR−Cas9 CD33-deleted allogeneic hematopoietic cell transplantation with gemtuzumab ozogamicin maintenance in AML: a phase 1/2 trial. *Nature Medicine* **2026**, *32*, 1763–1772, doi:10.1038/s41591-026-04362-1.
34. Luznik, L.; Pasquini, M.C.; Logan, B.; Soiffer, R.J.; Wu, J.; Devine, S.M.; Geller, N.; Giralt, S.; Heslop, H.E.; Horowitz, M.M.; et al. Randomized Phase III BMT CTN Trial of Calcineurin Inhibitor-Free Chronic Graft-Versus-Host Disease Interventions in Myeloablative Hematopoietic Cell Transplantation for Hematologic Malignancies. *J Clin Oncol* **2022**, *40*, 356–368, doi:10.1200/jco.21.02293.
35. Lum, S.H.; Albert, M.H.; Gilbert, P.; Sirait, T.; Algeri, M.; Muratori, R.; Fournier, B.; Laberko, A.; Karakukcu, M.; Unal, E.; et al. Outcomes of HLA-mismatched HSCT with TCRαβ/CD19 depletion or post-HSCT cyclophosphamide for inborn errors of immunity. *Blood* **2024**, *144*, 565–580, doi:10.1182/blood.2024024038.

36. Nalla, A.R.; Ganesan, K.; Muthukumar, V.; Balwani, M.; Seshadri, N.; Krishnakumar, K.; Uppuluri, R.; Raj, R. TCR-αβ/CD 19 Depleted Graft Versus T Replete Graft With Post-Transplant Cyclophosphamide in Children Undergoing Haploidentical Donor Hematopoietic Stem Cell Transplantation for Inborn Errors of Immunity: A Comparative Analysis From India. *Pediatr Transplant* **2026**, *30*, e70436, doi:10.1111/petr.70436.
37. Arcuri, L.J.; Natal, V.H.G.; Américo, A.D.; Perini, G.; Kerbauy, M.N.; Kerbauy, L.N.; Lima, A.C.M.; De León, A.G.; Ribeiro, A.A.F.; de Souza Santos, F.P.; et al. Impact of Modern Strategies for Graft-Versus-Host Disease Prophylaxis on Relapse Risk in Matched-Sibling or Unrelated Donor Hematopoietic Cell Transplantation: A Systematic Review and Meta-Analysis of Randomized Trials. *Eur J Haematol* **2026**, *117*, 432–441, doi:10.1111/ejh.70188.
38. Bentolila, G.; Desai, N.; Holtan, S.; Bonifazi, F.; Broers, A.E.C.; Mattsson, J.; Rolon, J.M.; Schroeder, T.; Kröger, N.; Gagelmann, N. A Network Meta-Analysis of Randomized Trials to Determine Optimal GVHD Prophylaxis in Matched Sibling Donor Transplantation. *Transplant Cell Ther* **2026**, *32*, 1028.e1-1028.e13, doi:10.1016/j.jtct.2026.05.004.
39. Jin, X.; Yang, Y.; Chen, X. Post-transplant cyclophosphamide versus anti-thymocyte globulin in haploidentical stem cell transplantation: a systematic review and meta-analysis. *Ann Hematol* **2025**, *104*, 1317–1328, doi:10.1007/s00277-025-06199-z.
